# Formative pluripotent stem cells show features of epiblast cells poised for gastrulation

**DOI:** 10.1038/s41422-021-00477-x

**Published:** 2021-02-19

**Authors:** Xiaoxiao Wang, Yunlong Xiang, Yang Yu, Ran Wang, Yu Zhang, Qianhua Xu, Hao Sun, Zhen-Ao Zhao, Xiangxiang Jiang, Xiaoqing Wang, Xukun Lu, Dandan Qin, Yujun Quan, Jiaqi Zhang, Ng Shyh-Chang, Hongmei Wang, Naihe Jing, Wei Xie, Lei Li

**Affiliations:** 1 State Key Laboratory of Stem Cell and Reproductive Biology, Innovation Academy for Stem Cell and Regeneration, Beijing Institute for Stem Cell and Regenerative Medicine, Institute of Zoology, University of Chinese Academy of Sciences, Chinese Academy of Sciences, Beijing, 100101 China; 2 Department of Cell Biology and Genetics, School of Basic Medical Sciences, Chongqing Medical University, Chongqing, 400016 China; 3 State Key Laboratory of Cell Biology, CAS Center for Excellence in Molecular Cell Science, Shanghai Institute of Biochemistry and Cell Biology, Chinese Academy of Sciences, Shanghai, 200031 China; 4 Center for Stem Cell Biology and Regenerative Medicine, MOE Key Laboratory of Bioinformatics, THU-PKU Center for Life Sciences, School of Life Sciences, Tsinghua University, Beijing, 100084 China; 5 Guangzhou Regenerative Medicine and Health Guangdong Laboratory (GRMH-GDL), Guangzhou Institutes of Biomedicine and Health, Chinese Academy of Sciences, Guangzhou, Guangdong 510530 China

**Keywords:** Embryonic stem cells, Embryonic stem cells

## Abstract

The pluripotency of mammalian early and late epiblast could be recapitulated by naïve embryonic stem cells (ESCs) and primed epiblast stem cells (EpiSCs), respectively. However, these two states of pluripotency may not be sufficient to reflect the full complexity and developmental potency of the epiblast during mammalian early development. Here we report the establishment of self-renewing formative pluripotent stem cells (fPSCs) which manifest features of epiblast cells poised for gastrulation. fPSCs can be established from different mouse ESCs, pre-/early-gastrula epiblasts and induced PSCs. Similar to pre-/early-gastrula epiblasts, fPSCs show the transcriptomic features of formative pluripotency, which are distinct from naïve ESCs and primed EpiSCs. fPSCs show the unique epigenetic states of E6.5 epiblast, including the super-bivalency of a large set of developmental genes. Just like epiblast cells immediately before gastrulation, fPSCs can efficiently differentiate into three germ layers and primordial germ cells (PGCs) in vitro. Thus, fPSCs highlight the feasibility of using PSCs to explore the development of mammalian epiblast.

## Introduction

Following implantation into the uterus at around embryonic day 3.5–4.5 (~E3.5–4.5), mouse amorphous epiblast cells transform into polarized epithelial cells and form a cup-shaped egg cylinder after being stimulated by Wnt/β-catenin, Fgf2/Erk and Activin signaling pathways.^1–3^ At ~E5.5, an anterior–posterior axis is established when the anterior visceral endoderm (AVE) is generated from the distal visceral endoderm (DVE) in mouse embryos.^4^ Starting from ~E6.0, epiblast cells rapidly proliferate and accumulate at the posterior area of egg cylinder to form the primitive streak, the hallmark of mammalian gastrulation.^5^ During gastrulation, epiblast cells migrate out from the primitive streak to form the mesendoderm (progenitors of endoderm and mesoderm) through an epithelial-to-mesenchymal transition (EMT), whereas the remaining epiblast cells develop into ectoderm.^6^ At ~E6.0, epiblast cells also differentiate into primordial germ cells (PGCs), the progenitors of male and female gametes, after being triggered by Bmp4 secreted from the extra-embryonic ectoderm.^7^ These early-to-late epiblast transitions are pivotal for cell fate specification and for establishing the foundation of the embryonic body plan in mammals.

Studies of the epiblast development of mammalian early postimplantation are extremely challenging, due to space-time constraints imposed by this highly dynamic process in uterus.^8^ Alternatively, mammalian epiblast cells may be maintained in vitro as stable cell lines, such as embryonic stem cells (ESCs) and epiblast stem cells (EpiSCs).^9–12^ As pluripotent stem cells (PSCs), these cell-lines can self-renew and differentiate into diverse functional cells in vitro, and show promising applications in regenerative medicine.^13,14^ Mouse ESCs (mESCs) are derived from pre-implantation embryos, whereas EpiSCs are obtained from post-implantation mouse embryos.^9,11,12^ mESCs can integrate into the inner cell mass (ICM) after blastocyst injection and develop into every cell type of an embryo; whereas EpiSCs barely incorporate into the ICM and cannot survive unless cell apoptosis is inhibited.^15–17^ At the transcriptome level, mESCs are similar to E4.5 early epiblast cells, whereas EpiSCs are similar to the anterior part of the E7.5 primitive streak.^18,19^ Thus mESCs were generally thought to correspond to naïve pluripotency (especially when cultured in 2i/lif conditions) with the broadest developmental potential of an embryo, whereas EpiSCs were thought to correspond to primed pluripotency with some pre-commitment to lineage specification.^20^ However, the process of cell lineage specification of ESCs and EpiSCs was found to be more complicated than initially thought. For example, mESCs cannot be efficiently induced into PGCs unless they were first converted into a metastable type of epiblast-like cells (EpiLCs), while EpiSCs are almost devoid of the capacity for PGC formation in vitro.^21,22^ On the other hand, in vivo, although their PGC differentiation potency is evanescent, pre-/early-gastrula epiblast cells show uniformly high potential for differentiation into all cell lineages of an embryo.^7^ It is possible that mESCs are immature to execute the induction cues for PGC formation, while EpiSCs are already fate-biased and thus lack the innate epiblast pluripotency that is indispensable for PGC differentiation. Thus, the naïve and primed state may not be sufficient to represent the full continuum of epiblast pluripotent states during mammalian early postimplantation development.^23,24^

Although tremendous efforts have been made to differentiate ESCs and EpiSCs into various types of functional cells for the clinical applications, the low efficiency and heterogeneity of directed differentiation remain as major technological barriers.^13^ Accumulating evidence suggest that mammalian epiblast cells may possess a series of intermediate pluripotent states between naïve and primed phase. For instance, the metastable EpiLCs induced from mESCs are intermediate pluripotent cells that are similar to E5.5 epiblast cells at the transcriptome level.^21^ Other intermediate PSCs have been derived from ESCs, suggesting multiple intermediate pluripotent states between naïve and primed state.^25–28^ For example, the “formative” states of intermediate pluripotency are proposed to be essential phases for epiblast cells to prepare transcriptomic, epigenomic, signaling and metabolic programs comprehensively for lineage specification following gastrulation.^23,24^ Because pre-/early-gastrula epiblast cells are the immediate progenitors that can differentiate into all cell types of an embryo, including PGCs and three germ layers, these cells may manifest the formative state of pluripotency.^23,24^ Such formative PSCs would not only illuminate the black box of epiblast maturation during mammalian implantation, but would also be invaluable for translational applications. Thus it is urgent to establish stable pluripotent stem cell lines for mammalian pre-/early gastrula epiblast cells to investigate the features of formative pluripotency.

In this current study, we have established formative pluripotent stem cells (fPSCs) with long-term self-renewal capacities from mouse ESCs, iPSCs and E5.5–6.5 epiblasts, through mimicking the in vivo environment of postimplantation developing embryos combined with suppression of the Wnt/β-catenin pathway. Comprehensive analyses show that fPSCs represent a stable intermediate state between naïve ESCs and primed EpiSCs, with the unique features of transcriptome, epigenome and differentiation potency, similar to that of mouse epiblast cells just before gastrulation, and poised for differentiation into all embryonic cell lineages including PGCs.

## Results

### Generation of intermediate state Epiblastoids from mESCs

**Fig. 1:**
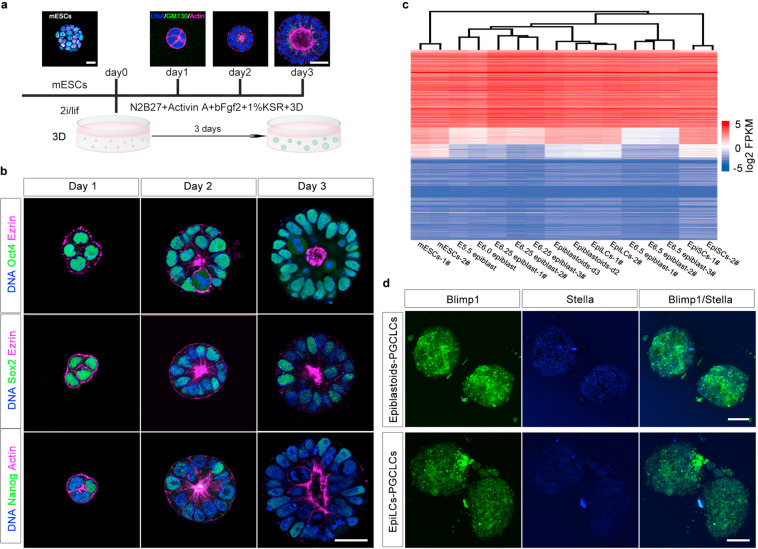
Generation of intermediate state Epiblastoids from mESCs. **a** The generation of Epiblastoids from mESCs. Epiblastoids were self-organized and differentiated from naïve mESCs in a 3D system with EpiLC formation conditions for 1–3 days and were stained for GM130 (green), F-actin cytoskeleton (Phalloidin, magenta), and DNA (Hoechst 33342, blue). Scale bars, 20 μm. **b** Immunostaining analysis of the expression of Oct4/Pou5f1, Sox2, and Nanog (green) during the transition from mESCs to Epiblastoids at days 1, 2, and 3. Apical lumen was labeled by Ezrin or F-actin staining (magenta). DNA was stained with Hoechst 33342 (blue). Scale bars, 20 μm. **c** Hierarchical clustering analysis was performed for RNA-seq data of mESCs, 48 h EpiLCs, Epiblastoids at days 2 and 3 (Epiblastoids-d2, -d3), EpiSCs and epiblast cells. The data of E5.5, E6.0, E6.25, and E6.5 epiblast were obtained from previous reports^30–32^ with the transcriptome of all genes among these cells. -1#, -2#, and -3# represented different sample repeats. **d** Epiblastoids and EpiLCs were induced into PGCLCs as previously reported.^21^ After differentiation of 6 days, Blimp1^+^ (green) and Stella^+^ (blue) cells displayed in the PGCLC aggregates of Epiblastoids and EpiLCs, and photographed with confocal microscope. Scale bars, 100 μm.

To derive the intermediate state cells, we cultured CMTI-1 mESCs in a three dimensional (3D) system to mimic the in vivo environment of developing embryos with EpiLC formation conditions (Fig. 1a).^21^ mESCs polarized and self-organized in 3D culture to form epiblast-like embryoids (hereinafter referred to as Epiblastoids), defined by the polarized localization of F-actin and GM130, an apical marker (Fig. 1a), consistent with a previous report.^29^ Epiblastoids developed a lumen at the center after 2 days of differentiation (Fig. 1a). The polarization and lumen formation of Epiblastoids was also confirmed by the staining of Ezrin, another apical marker and a member of the ERM (Ezrin/Radixin/Moesin) family (Fig. 1b). During the differentiation of mESCs into Epiblastoids, Oct4/Pou5f1 was expressed persistently and homogenously, whereas Sox2 expression developed into a mosaic pattern after 2–3 days of differentiation (Fig. 1b). The Nanog expression pattern also became heterogeneous, but decreased more sharply as early as day 1 after differentiation (Fig. 1b).

RNA sequencing (RNA-seq) was performed for mESCs (mESCs-1, -2), Epiblastoids at day 2 and 3 (Epiblastoids-d2, -d3), 48 h EpiLCs (EpiLCs-1, -2) and EpiSCs (EpiSCs-1, -2), and these data were compared with the published data of E5.5–6.5 epiblast cells in vivo.^30–32^ Unsupervised hierarchical clustering analysis using genome-wide gene expression and differentially expressed genes (DEGs) suggested that the cells of Epiblastoids-d2 and -d3 were similar to 48 h EpiLCs and E5.5–6.5 epiblasts, but distinct from mESCs or EpiSCs (Fig. 1c; Supplementary information, Fig. S1a). Epiblastoids-d2 and -d3 were similar to the late stage EpiLCs of 72–96 h differentiation (Supplementary information, Fig. S1b).^25,33^ Then we tried to induce BVSC mESCs into Blimp1-Stella reporter Epiblastoids to investigate their differentiation potential for PGCs. Blimp1-Stella reporter Epiblastoids did not express Blimp1/Stella. After differentiated into PGC-like cells (PGCLCs) as previously reported,^21^ Blimp1^−^/Stella^−^ Epiblastoids showed the capacity to differentiate into PGCLCs (Blimp1^+^/Stella^+^) cells, similar to metastable EpiLCs (Fig. 1d). All together, these data suggest that the Epiblastoids represent an alternative pluripotent state distinct from mESCs and EpiSCs, probably similar to ~E5.5–6.5 epiblast cells.

### Stabilizing and maintaining Epiblastoids by inhibiting Wnt/β-catenin activity

**Fig. 2:**
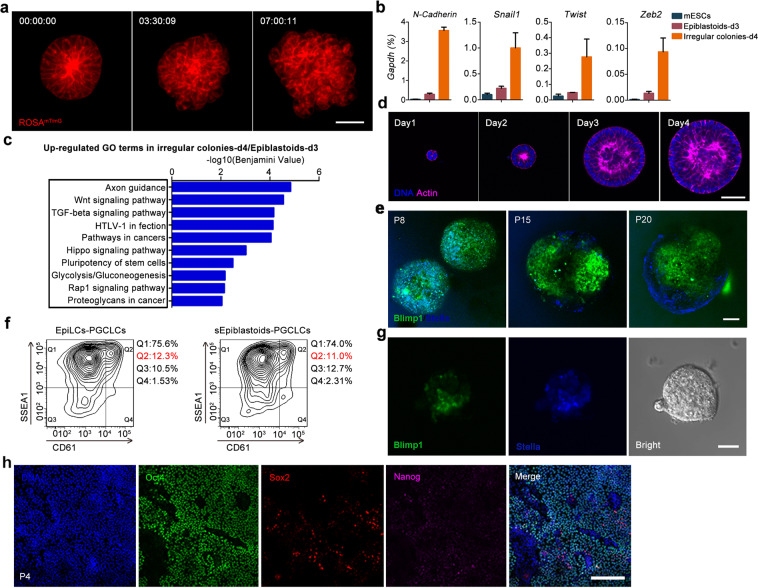
Stabilizing and maintaining Epiblastoids by inhibiting Wnt/β-catenin activity. **a** After culture for 3 days, *ROSA*^*mT/mG*^ Epiblastoids (red) were recorded every 15 min for 24 h with Ultra VIEW-Vox system. Time-lapse images were processed with Velocity software 6.0. Scale bar, 40 μm. **b** qRT-PCR analysis of EMT-related markers *N-cadherin*, *Snail1, Twist*, and *Zeb2* in mESCs, Epiblastoids at day 3 (Epiblastoids-d3), and irregular colonies at day 4 (irregular colonies-d4). The level of specific gene expression was normalized to *Gapdh* (100%). Data were represented as means ± SEM (*n* = 3). **c** GO analysis showed that the upregulated genes in day 4 irregular colonies were enriched in Wnt, TGF-beta and Hippo signaling pathways, compared with those in day 3 Epiblastoids. **d** mESCs differentiated into metastable Epiblastoids and were maintained in the medium with XAV939 (Epiblastoids medium) for 4 days. The different stages of cells were stained with Phalloidin (F-actin, magenta) and Hoechst 33342 (DNA, blue). Scale bar, 40 μm. **e** The Blimp1^+^ (green) and Stella^+^ (blue) cells displayed in the PGCLC aggregates of passage 8 (P8), 15 (P15) and 20 (P20) of Blimp1*/*Stella reporter sEpiblastoids at day 6 of differentiation. Scale bar, 100 μm. **f** FACS analysis of SSEA1 and CD61 (integrin-β3) double-positive cells for the aggregates of PGCLCs induced from P8-sEpiblastoids and EpiLCs at day 6 after differentiation. **g** PGCLCs (Blimp1^+^ and Stella^+^) displayed in the intact colonies of sEpiblastoids at day 6 after differentiation. Scale bars, 100 μm. **h** EpiLCs were induced from mESCs and propagated in sEpiblastoid medium in 2D culture condition. The immunofluorescent staining of Oct4, Sox2 and Nanog was performed for EpiLCs of passage 4 (P4). Scale bar, 200 μm.

The Epiblastoids lost their polarity and formed irregular colonies when they were propagated in the same conditions (Supplementary information, Fig. S2a). Similarly, metastable EpiLCs could not be maintained as a stable cell line in these conditions as previously reported (Supplementary information, Fig. S2b).^21^ To better understand Epiblastoids formation after extended culturing, we generated Epiblastoids by using *ROSA*^*mT/mG*^ mESCs.^34,35^ Time-lapse microscopy showed that after 3 days of culture, the polarity of Epiblastoids was rapidly destroyed within ~3 h, and some cells migrated out from the well-defined colonies at ~7 h (Fig. 2a; Supplementary information, Video S1), reminiscent of EMT during gastrulation. Quantitative RT-PCR (qRT-PCR) analysis showed that the EMT markers, *N-Cadherin, Snail1*, *Twist*, and *Zeb2* were all induced at day 4 (Fig. 2b), supporting the notion that EMT occurred after 3 days of culture. RNA-seq and gene ontology (GO) enrichment analysis showed that day-4 cells upregulated the signatures for Wnt/β-catenin, TGF-β, and Hippo-Yap pathways relative to day-3 cells (Fig. 2c), all of which are important regulators of EMT during gastrulation.

Next, we sought to investigate if we could prevent the collapse of Epiblastoids after propagation, by adding a Wnt/β-catenin inhibitor (XAV939), a TGF-β inhibitor (SB431542) or a Yap inhibitor (Verteporfin). Treatment with all these inhibitors did not affect the apical lumen formation of Epiblastoids (Supplementary information, Fig. S2c–e). However, when these cells were passaged, only XAV939 treatment maintained the formation of an apical lumen in Epiblastoids (Supplementary information, Fig. S2c), whereas SB431542 treatment led to the loss of cell polarity and Verteporfin treatment caused severe cell death (Supplementary information, Fig. S2d, e). Moreover, when supplemented with XAV939, the metastable Epiblastoids could be maintained as polarized and cavitated 3D structures for more than 3 days (Fig. 2d), and propagated for long-term culture in these 3D conditions (Fig. 2e).

XAV939 inhibits the poly-ADP-ribosylating enzymes to stabilize axin2, thus stimulating β-catenin degradation and inhibiting the activity of Wnt/β-catenin pathway.^36^ To investigate the specific role of XAV939 in the formation of Epiblastoids, we tried to generate Epiblastoids using *Ctnnb*^*(ΔEX3/+)*^ mESC,^37^ in which the truncated β-catenin is prevented from degradation (stabilized) and therefore constitutively active.^38^ Our results showed that Epiblastoids could not be established from *Ctnnb*^*(ΔEX3/+)*^ mESCs, which developed into a solid sphere rather than a polarized and cavitated rosette 3D structure, even in the presence of XAV939 (Supplementary information, Fig. S2f). These results suggest that specific inhibition of Wnt/β-catenin activity is critical for Epiblastoid stabilization.

As the culture medium also included Activin A and Fgf2, we further investigated the role of these growth factors in the formation and maintenance of Epiblastoids. Supplemented with XAV939, Epiblastoids formed the apical lumen in polarized 3D structures when the medium was deprived of Activin A and/or Fgf2 (Supplementary information, Fig. S2g). However after passaging, even in the presence of ROCK inhibitor Y-27632, the cells barely survived without both Activin A and Fgf2, and lumen formation was compromised in the absence of any Activin A or Fgf2 (Supplementary information, Fig. S2g). These data suggest that both Activin A and Fgf2 are necessary for the continual survival and growth of the stabilized Epiblastoids (sEpiblastoids).

Notably, similar to metastable EpiLCs and Epiblastoids, the sEpiblastoids could efficiently differentiate into the progenitors of germ cells (Fig. 2e). In PGCLC differentiation conditions, *Blimp1-Stella*-derived sEpiblastoids generated ~56% Blimp1^+^ cells at day 2 (Supplementary information, Fig. S2h); by day 6, these cells were induced into PGCLCs (CD61^+^ and SSEA1^+^) at an efficiency of ~11%, comparable to that of EpiLCs (~12%) (Fig. 2f). Furthermore, long-term propagation barely affected the gametogenetic capacity of sEpiblastoids even after 20 passages (Fig. 2e). In addition, sEpiblastoids could also be induced into PGCLCs as intact clones (Fig. 2g), similar to the induction of PGCLCs from mouse epiblast cells.^7^ Although the medium supplemented with XAV939 could facilitate the propagation of EpiLCs in 2D culture conditions for at least 4 passages that expressed Oct4 and Sox2 (Fig. 2h), the propagated EpiLCs experienced severe cell death and could not aggregate well when they were induced to PGCLCs (Supplementary information, Fig. S2i). In sum, these data suggest that the Epiblastoids can undergo stabilization and long-term self-renewal through specific inhibition of Wnt/β-catenin pathway.

### Stabilized Epiblastoids possess features of fPSCs

**Fig. 3:**
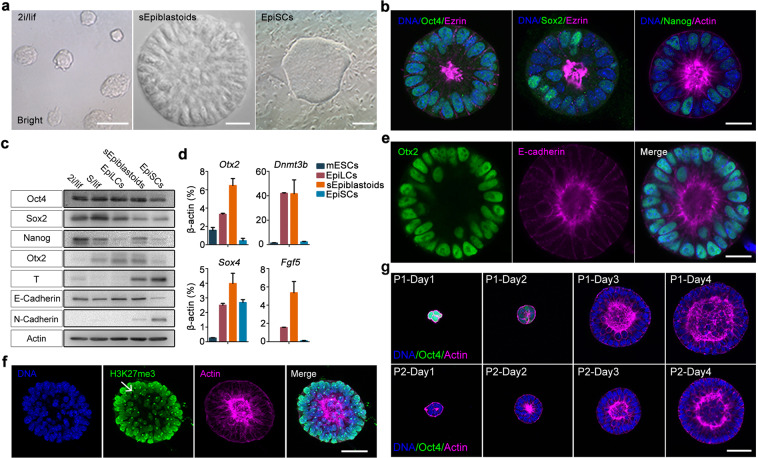
Stabilized fPSCs possess features of formative pluripotent state. **a** The morphology of typical naïve mESCs (left panel), sEpiblastoids (middle panel), and EpiSCs (right panel); scale bars, 50 μm (left panel), 20 μm (middle panel), and 300 μm (right panel). **b** The immunofluorescent staining of sEpiblastoids for Oct4, Sox2, or Nanog (green) and Ezrin or F-actin (magenta). DNA was stained with Hoechst 33342 (blue). Scale bar, 20 μm. **c** The lysates of mESCs (2i/lif or S/lif), 48 h EpiLCs, sEpiblastoids and EpiSCs were collected, and analyzed by western blot assay with specific antibodies for Oct4, Sox2, Nanog, Otx2, T, E-Cadherin and N-Cadherin. β*-*actin served as the loading control. **d** The expression of putative formative pluripotency markers (*Otx2*, *Dnmt3b, Sox4* and *Fgf5*) was examined for naïve mESCs, EpiLCs, sEpiblastoids and EpiSCs by qRT-PCR with specific primers of these genes. The gene expression level was measured relative to *β-actin* (100%). Data were represented as means ± SD (*n* = 3). **e** sEpiblastoids were stained with specific antibodies for Otx2 (green), E-Cadherin (magenta), and Hoechst 33342 for DNA (blue). Scale bar, 20 μm. **f** Female fPSCs were stained with specific antibody for H3K27me3 (green) and Phalloidin for F-actin (magenta). DNA was stained with Hoechst 33342 (blue). Scale bar, 40 μm. **g** *Oct4-ΔPE-GFP* reporter mESCs were cultured in fPSC medium for 4 days (fPSCs passage 1, P1, top). P1 cells were propagated (P2, bottom) in the same medium. The cells of days 1–4 were stained with Phalloidin for F-actin (magenta) and Hoechst 33342 for DNA (blue). The enhancer utilization of *Oct4* was indicated by GFP reporter (green). Scale bar, 40 μm.

We then sought to characterize the sEpiblastoids. Morphologically, sEpiblastoids grew into polarized and cavitated 3D structures with smooth margins, distinct from naïve mESCs (small, domed colonies) and EpiSCs (big, flat colonies) (Fig. 3a). The cells of sEpiblastoids displayed a typical exponential growth model and had a normal karyotype (Supplementary information, Fig. S3a, b). The colony formation efficiency of sEpiblastoids (~40%) was lower than that of naïve mESCs (~50%), but much higher than that of EpiSCs (~15%) when propagated as single cells as previously reported (Supplementary information, Fig. S3c).^39^ The staining of alkaline phosphatase (AP) in sEpiblastoids was similar to that in mESCs, but stronger than that in EpiSCs (Supplementary information, Fig. S3d).

Similar to the unstable Epiblastoids, protein expression of the pluripotency marker Oct4 was detected in all cells of sEpiblastoids, with the level comparable to that in mESCs and EpiLCs, but higher than that in EpiSCs (Fig. 3b, c). The expression of Sox2 and Nanog protein displayed a mosaic pattern in sEpiblastoids (Fig. 3b). Compared with mESCs, sEpiblastoids expressed low levels of Sox2 protein, similar to those in EpiSCs (Fig. 3c). The protein level of Nanog in sEpiblastoids was between those in naïve (2i/lif) mESCs and primed EpiSCs (Fig. 3c). Compared with EpiSCs, sEpiblastoids expressed high levels of E-Cadherin, but low levels of N-Cadherin and primed/lineage-specific markers (Brachyury/T, Gata6, and Foxa2) (Fig. 3c; Supplementary information, Fig. S3e). Interestingly, compared with mESCs and EpiSCs, sEpiblastoids specifically expressed higher levels of Otx2/*Otx2*, a proposed marker of formative PSCs (Fig. 3c–e).^23,24^ sEpiblastoids also highly expressed other proposed formative markers *Dnmt3b*, *Sox4*, and *Fgf5* (Fig. 3d). Based on their expression of formative pluripotency markers and long-term self-renewal, sEpiblastoids are referred to as formative pluripotent stem cells (fPSCs) hereafter.

Naïve mESCs exhibit two active X chromosomes (XaXa) in female cells and preferentially utilize the distal enhancer of *Oct4*, whereas female EpiSCs show inactivation on one of the X chromosomes (XaXi) and use the proximal enhancer of *Oct4*.^40^ To examine the X chromosome state, female fPSCs were established from female mESCs and stained for H3K27me3 as previously reported.^41^ Our results suggested that one X chromosome was inactive in the fPSCs (XaXi), evidenced by the formation of H3K27me3 foci (Fig. 3f). Next, the utilization of *Oct4* enhancer in fPSCs was also investigated with an *Oct4-ΔPE-GFP* reporter mESCs.^40^ Our results showed that distinct from mESCs, the distal enhancer of *Oct4* became inactive shortly after mESCs initiated differentiation in the first passage, and it was no longer used in subsequent passages (Fig. 3g). Therefore, fPSCs share similar X chromosome inactivation and *Oct4* enhancer utilization with EpiSCs, but not with mESCs.

### fPSCs are transcriptomically similar to in vivo E5.5–6.5 epiblasts

**Fig. 4:**
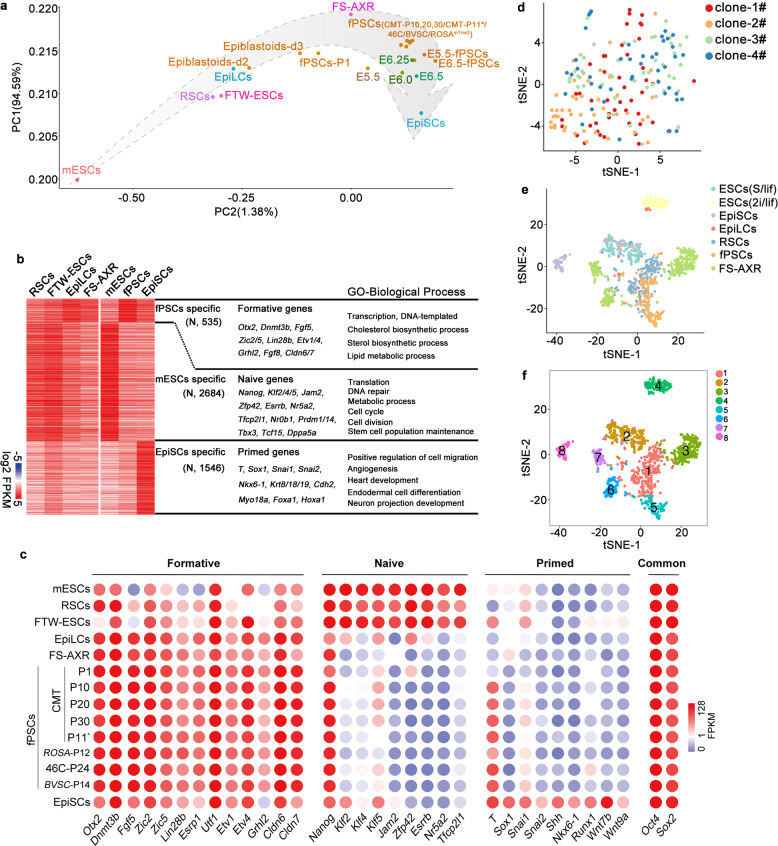
Self-renewing fPSCs are transcriptomically similar to early gastrula epiblasts. **a** Principle component analysis (PCA) of naïve mESCs, EpiSCs, EpiLCs, RSCs,^42^ FTW-ESCs,^44^ FS-AXR,^43^ Epiblastoids-d2/3, fPSCs-P1 (CMT passage 1), fPSCs (CMT-P10, 20, 30/CMT-P11*/46 C/*BVSC*/*ROSA*^*mT/mG*^), E5.5-, E6.5-fPSCs, and E5.5–E6.5 (E5.5–E6.5 epiblast cells).^30–32^ CMT-P11*, cryopreserved passage 11 of the fPSCs derived from CMT; fPSCs (46 C/*BVSC*/*ROSA*^*mT/mG*^), passage 24/14/12; E5.5-, E6.5-fPSCs, fPSCs derived from mouse E5.5 and 6.5 epiblasts. **b** Heatmap of the highly expressed genes (*N*, number of genes) in naïve mESCs, EpiSCs and fPSCs (fold change ≥ 1.5, FPKM value ≥ 3). Representative genes of different clusters were selected and listed in the middle. GO enrichment term results of the highly expressed genes in fPSCs, mESCs and EpiSCs were shown on the right. The expression of these specific genes in RSCs, FTW-ESCs, 48 h EpiLCs and FS-AXR were also shown in the heatmap. **c** The gene expression of selected formative, primed, naïve and common pluripotent markers in mESCs, RSCs, FTW-ESCs, EpiLCs, FS-AXR, fPSCs (different passages and background), and EpiSCs detected by RNA-seq. **d** t-SNE analysis of scRNA-seq data of fPSCs. Clone1/2/3/4# represented four independent clones of fPSCs-Passage11 (fPSCs-P11). **e** Data integration and t-SNE analysis of fPSC single cells with previous data of ESCs (2i/lif, Serum/lif), EpiLCs (48 h), RSCs, FS-AXR and EpiSCs.^42,43,53,54^ The originating cell types were labeled with different colors. **f** The integrated data of mESCs (2i/lif, Serum/lif), EpiLCs (48 h), RSCs, FS-AXR, fPSCs, and EpiSCs were further clustered into eight cell populations through t-SNE analysis. Different cell populations were represented by different colors (cluster-1 to -8).

To investigate the genome-wide gene expression, RNA-seq was performed on the fPSCs at different passages of CMT (P1, 10, 20, 30) and different background derived from 46C, BVSC and *ROSA*^*mT/mG*^ mESCs. We compared these data with previous data, as well as the data of recently reported intermediate PSCs, such as RSCs (Rosette-like stem cells),^42^ FS-AXR^43^ and FTW-ESCs.^44^ 2D-principal component analysis (2D-PCA) suggested that the long term propagated fPSCs and FS-AXR were most similar to E6.0–6.5 epiblasts (Fig. 4a). Hierarchical clustering of RNA-seq suggested that fPSCs were close to 48 h EpiLCs, FS-AXR, EpiSCs and in vivo epiblasts, whereas RSCs and FTW-ESCs were closer to mESCs (Supplementary information, Fig. S4a). When compared with the EpiLCs at different stages, fPSCs were closer to the EpiLCs at late stage of differentiation (Supplementary information, Fig. S4b).^25^ The fPSCs cryopreserved or derived from different backgrounds (CMTI-1, 46 C, BVSC and *ROSA*^*mT/mG*^ mESCs) had a similar transcriptome (Fig. 4a; Supplementary information, Fig. S4a). Based on deconvolution analysis of the spatial pattern of transcriptomic features,^45^ fPSCs and FS-AXR were closest to epiblast cells at ~E6.0–6.5, while RSCs and FTW-ESCs were closest to epiblast cells at ~E5.5–6.0, and mESCs and EpiSCs were most similar to E4.5 ICM and some E7.0 epiblast cells, respectively (Supplementary information, Fig. S4c).

To investigate specific genes that distinguish fPSCs from EpiSCs and mESCs, we performed differential gene expression analysis for these cells. In total, 4765 DEGs were identified amongst fPSCs, mESCs and EpiSCs, based on the criterion that the gene’s expression level in one cell-type higher than that in the other two cell-types (Fold change ≥ 1.5, FPKM value ≥ 3) (Fig. 4b). Compared with mESCs and EpiSCs, 535 genes were highly expressed in fPSCs, including many formative genes such as *Dnmt3b*, *Otx2*, and *Fgf5* (Fig. 4b, c; Supplementary information, Table S1). GO analysis revealed that many genes highly expressed in fPSCs were involved in cholesterol/sterol biosynthetic process and lipid metabolic process (Fig. 4b), supporting the role of lipid metabolism in the maintenance of intermediate state PSCs.^46,47^ The genes upregulated in fPSCs also included the transcription factor *Zic2/5*, *Etv1/4* and *Grhl2*, which were reported to play important roles in the transition from the naïve to primed state,^26,48,49^ and several well-known regulators of pluripotency such as *Lin28b*, *Utf1*, *Zscan10*, *Fgf8*, *Eras*, as well as epithelial regulators *Esrp1*, *Cldn6*/*7*,^37,50–52^ suggesting that these genes could be new markers for formative-like pluripotency (Fig. 4b, c). In comparison, genes upregulated in mESCs included a suite of well-known naive pluripotency markers such as *Nanog*, *Klf2,4,5*, *Jam2*, *Zfp42/Rex1*, *Esrrb*, *Nr5a2*, and *Tfcp2l1* (Fig. 4b, c; Supplementary information, Table S2). In contrast, genes upregulated in EpiSCs (relative to fPSCs and mESCs) were enriched in lineage-specific genes, including numerous signatures for angiogenesis, heart development, endodermal and nervous system differentiation such as *T*, *Sox1*, *Snai1*, *2*, *Shh*, *Nkx6-1*, *Runx1*, *Wnt7b* and *Wnt9a* (Fig. 4b, c; Supplementary information, Table S3). Probably, the expression patterns of these specific genes in RSCs, FTW-ESCs, EpiLCs and FS.AXR were consistent with the continuum of PSC differentiation (Fig. 4b, c). Quantitative RT-PCR confirmed the expression of formative, naïve and lineage-specific genes in mESCs, 48 h EpiLCs, fPSCs and EpiSCs (Supplementary information, Fig. S4d–f).

We also performed the single cell analysis for fPSCs. Totally, 192 cells were obtained from 4 clones (4 × 48) for single-cell RNA sequencing (scRNA-seq). An average of 3721 genes was detected per cell, suggesting a relatively good sequencing quality (Supplementary information, Fig. S5a, b). T-distributed stochastic neighbor embedding (t-SNE) analysis with stringent parameters suggested that there are two sub-populations among these single cells (Fig. 4d and Supplementary information, Fig. S5c). However, we could only identify 6 DEGs among these single cells (Supplementary information, Fig. S5d). Then, we compared these data with the published single cell data of mESCs (2i/lif, Serum/lif), RSCs, EpiLCs (48 h), FS-AXR and EpiSCs.^42,43,53,54^ t-SNE plot showing that all of these single cells were grouped into 8 clusters, including naïve PSCs (cluster-4) and primed PSCs (cluster-8) (Fig. 4e, f; Supplementary information, Fig. S5e–f and Table S4). fPSCs were mostly distributed in cluster-1 and -5 with no significant DEGs identified, and FS-AXR were mainly distributed in cluster-3 with 18 DEGs, cluster-6 with 35 DEGs and cluster-7 with 45 DEGs (Fig. 4e, f; Supplementary information, Fig. S5e, f and Table S4). Collectively, these gene expression data support the notion that fPSCs manifest formative pluripotency, similar to pre-/early-gastrula epiblasts, but distinct from mESCs and EpiSCs, also probably and likely different from other PSCs recently established (RSCs, FTW-ESCs, FS-AXR).

### fPSCs possess chromatin states similar to in vivo E6.5 epiblast cells

**Fig. 5:**
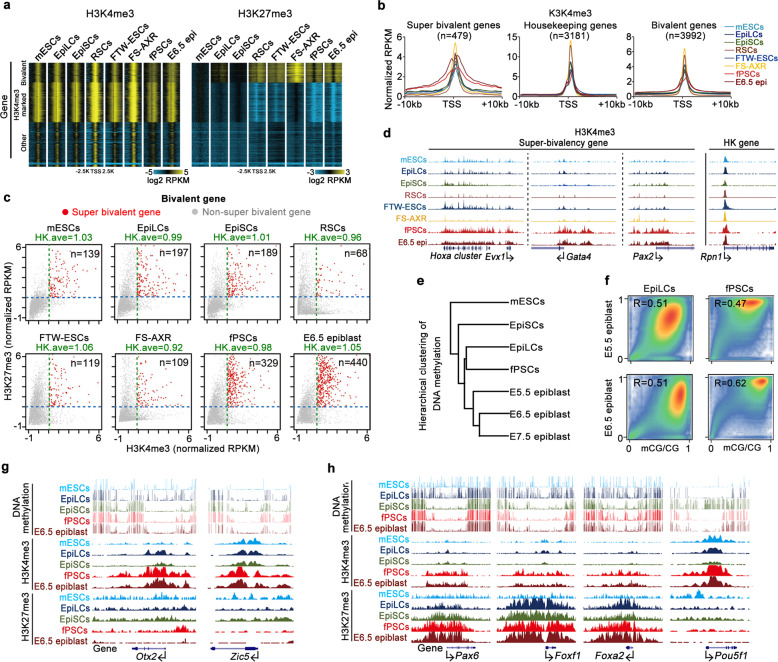
fPSCs possess similar chromatin states of E6.5 epiblasts. **a** Heatmaps showing H3K4me3/H3K27me3 enrichment at all promoters of genes (± 2.5 kb) in naïve mESCs, EpiLCs, EpiSCs, fPSCs, E6.5 epiblast and recently reported RSCs, FTW-ESCs as well as FS-AXR. The intensity of H3K4me3/H3K27me3 was normalized as RPKM. The data of H3K4me3/H3K27me3 for naïve mESCs, EpiLCs, EpiSCs, RSCs, FTW-ESCs, FS-AXR, and E6.5 epiblast were obtained from the published resources.^42–44,56,57^ **b** Average plot showing H3K4me3 enrichment at all loci of super-bivalent, housekeeping, and bivalent genes in naïve mESCs, EpiLCs, EpiSCs, RSCs, FTW-ESCs, FS-AXR, fPSCs, and E6.5 epiblast. The H3K4me3 enrichment at super bivalent and bivalent genes was normalized to the average intensity of housekeeping genes in each cell line. **c** Scatter plots displayed the enrichment of H3K4me3 and H3K27me3 of 3992 bivalent genes in different cell types. The average H3K4me3 enrichment of housekeeping genes (HK. ave) was shown for each of cell types. The blue lane indicate an arbitrary cutoff of H3K27me3 for each stage (normalized RPKM > 1). Super-bivalent and bivalent genes were shown as red and gray, respectively. The number of super-bivalent genes (right top) was shown in naïve mESCs, EpiLCs, EpiSCs, RSCs, fPSCs, FTW-ESCs, FS-AXR, and E6.5 epiblasts. **d** UCSC Genome Browser views showing H3K4me3 enrichment at the locus of super-bivalent genes (*Hoxa* cluster, *Gata4*, and *Pax2*) and housekeeping gene (*Rpn1*) in mESCs, EpiLCs, EpiSCs, RSCs, FTW-ESCs, FS-AXR, fPSCs, and E6.5 epiblast. **e** Hierarchical clustering analysis of DNA methylation for naïve mESCs, EpiLCs, fPSCs, EpiSCs and E5.5/6.5/7.5 epiblasts.^58–61^ **f** Smoothed scatter plot of genome-wide DNA methylation between EpiLCs or fPSCs and E5.5, or E6.5 epiblasts. Density of data pointed the ranges from blue (low) to green (intermediate) and yellow (high). **g** Genome Browser views showing the DNA methylation and H3K4me3/H3K27me3 enrichment at the promoters of selected formative markers (*Otx2*, *Zic5*) in naïve mESCs, EpiLCs, and EpiSCs, as well as fPSCs and E6.5 epiblasts. **h** Snapshots of DNA methylation and enrichment of H3K4me3 and H3K27me3 at the promoters of selected germ-layer markers (*Pax6*, *Foxf1* and *Foxa2*) and *Oct4* in naïve mESCs, EpiLCs, and EpiSCs, as well as fPSCs and E6.5 epiblasts.

Aside from the gene expression profiles at steady-state, developmental potential of PSCs are also tightly regulated by the epigenomic states of chromatin organization, as indicated by imprinting-related DNA methylation and histone modifications.^55^ fPSCs were proposed to display increased numbers of bivalent chromatin domains marked by both active H3K4me3 and repressive H3K27me3, as well as globally elevated DNA methylation.^23,24^ Thus, ChIP-seq of H3K4me3 and H3K27me3 was performed for fPSCs and compared with the published data of ESCs, RSCs, FTW-ESCs, EpiLCs, FS-AXR, EpiSCs, and the E6.5 epiblast.^42–44,56,57^ Globally, the distribution of H3K27me3 at the promoter regions of all genes was stronger in fPSCs, RSCs, FTW-ESCs, FS-AXR and the E6.5 epiblast than in other PSCs, while H3K4me3 distribution at the promoter regions of all genes displayed no discernible differences amongst these pluripotent cells (Fig. 5a). Unsupervised hierarchical clustering of H3K4me3 and H3K27me3 suggested that, amongst all these PSCs, fPSCs were the most similar to E6.5 epiblast cells (Supplementary information, Fig. S6a). Although the level of H3K27me3 was relatively low in EpiLCs, we could observe that H3K27me3 was enriched at many promoters of the shared bivalent genes when carefully compared with E6.5 epiblasts (Fig. 5a; Supplementary information, Fig. S6b, c).

We have previously reported a unique chromatin state that showed strong H3K4me3 and H3K27me3 enrichment at the promoters in a subset of bivalent genes in E6.5 epiblasts (over 400 developmental genes).^57^ The H3K4me3 distribution at the promoters of these genes is remarkably broader than typical bivalent promoters and the average promoter enrichment is even stronger than those of housekeeping genes, thus termed as super bivalency.^57^ Then super bivalency was closely analyzed in the promoters of all genes in these pluripotent cells. Compared with other PSCs, the H3K4me3 distribution in the promoter region of all bivalent and super bivalent genes was globally broader in fPSCs and the E6.5 epiblasts, while the H3K4me3 distributions for housekeeping genes was similar in all PSCs (Fig. 5b). The number of super bivalent genes was substantially higher in fPSCs than that in the RSCs, FTW-ESCs and FS-AXR, respectively (Fig. 5c; Supplementary information, Table S5). Furthermore, 89% (294/329) of the super bivalency genes in fPSCs were also present in the E6.5 epiblasts (Supplementary information, Fig. S6d). For example, the *Hoxa* cluster, *Gata4*, and *Pax2* showed super bivalency only in fPSCs and the E6.5 epiblasts, but not in other PSCs (Fig. 5d). GO analysis showed that these 329 super bivalent genes in fPSCs were involved in three germ layer specification, anterior/posterior pattern formation and embryonic morphogenesis (Supplementary information, Fig. S6e), suggesting that these genes are poised for the next stage in embryonic development.

Whole genome bisulfite sequencing (WGBS) was also performed on fPSCs and compared with the published datasets.^58–61^ Globally, fPSCs had high levels of DNA methylation at the gene body and intergenic region, similar to those in EpiSCs, EpiLCs and postimplantation epiblast cells, whereas naïve mESCs showed global hypomethylation (Supplementary information, Fig. S6f, g). Unsupervised hierarchical clustering of DNA methylation showed that both fPSCs and EpiLCs were the closest to in vivo epiblasts, compared to other PSCs (Fig. 5e). Smoothed scatter plot analysis suggested that fPSCs (*R* = 0.62) were more similar to the E6.5 epiblasts at global DNA methylation levels (Fig. 5f).

Specific genes were further investigated for correlations between their gene expressions and epigenetic states in these PSCs. Consistent with their high levels of gene expression, the promoters of formative genes (*Otx2*, *Zic5*, *Utf1*, *Fgf5*, *Zfp13*, *Zscan10*, *Zic2*, and *Esrp1*) were all marked by higher levels of the active marker H3K4me3, and low levels of the repressive marker H3K27me3 and hypo-methylated DNA in fPSCs, similar to E6.5 epiblast cells (Fig. 5g; Supplementary information, Fig. S7a). Promoters of lineage-specific genes (*Pax6*, *Foxf1*, *Foxa2*, *T*, *Gata6*, *Lhx5*, *Hand1, Isl1, Nkx2-5, Nr4a2* and *Pdx1*) displayed high levels of both H3K4me3 and H3K27me3, but low levels of DNA methylation in fPSCs and E6.5 epiblast cells, even though they are not highly expressed, suggesting that lineage-specific genes exist in the poised state in fPSCs and E6.5 epiblast cells (Fig. 5h; Supplementary information, Fig. S7b). Previous studies have revealed that many large hypomethylated regions (DNA methylation valleys, DMVs) are present near the promoter of developmental genes and may play important roles in the transcriptional plasticity of these gene during cell fate commitment.^62,63^ Interestingly, we also observed that the DMVs of three lineage-specific genes (*Pax6*, *Foxf1* and *Foxa2*) displayed aberrantly high DNA methylation levels in EpiLCs and that the DMV of the *Oct4* gene was highly methylated in EpiSCs, likely suggesting that they show biases in cell fate specification upon differentiation (Fig. 5h). Taken together, these results suggest that fPSCs are the closest to E6.5 epiblast cells at the epigenomic level, compared to other PSCs including RSCs, FTW-ESCs and FS-AXR.

### fPSCs efficiently differentiate into three germ layers in vitro

**Fig. 6:**
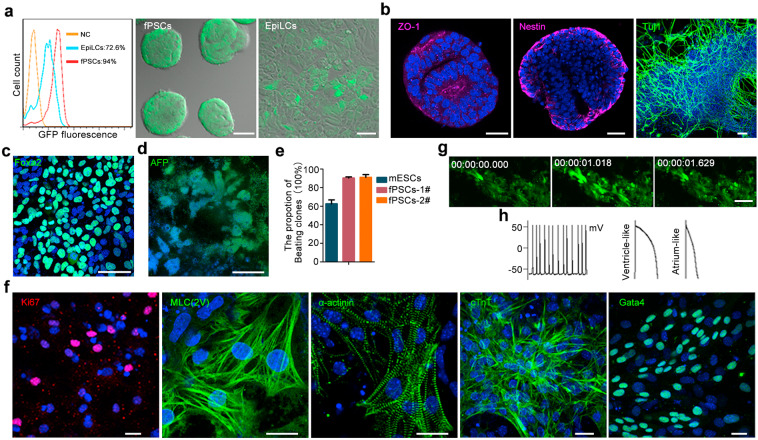
fPSCs efficiently differentiate into all cell lineages of embryo in vitro. **a** *Sox1-GFP* fPSCs and EpiLCs were derived from 46C mESCs and differentiated in neural induction conditions. After 2 days of differentiation, Sox1-GFP^+^ cells were analyzed by FACS. FACS analysis result was shown in left panel (NC, undifferentiated *Sox1-GFP* fPSCs). The photograph of Sox1-GFP^+^ cells were also shown. Scale bars, 100 μm (middle panel), 30 μm (right panel). **b** Neuronal lineage cells differentiated from fPSCs were stained with ZO-1 (tight junction protein 1, magenta) and Nestin (neural stem cell marker, magenta) antibodies at day 4, and Tuj1 (neuron marker, green) antibody at day 7 after differentiation. DNA was stained with Hoechst 33342 (blue). Scale bars, 50 μm. **c** fPSCs were induced into hepatocyte-like cells. At day 5 after differentiation, these cells were stained with Foxa2 (definitive endoderm marker, green). Nucleus was stained with Hoechst 33342. Scale bar, 50 μm. **d** The hepatocyte-like cells differentiated from fPSCs were stained with the antibody for AFP (liver cell marker, green) at day 12 after differentiation. DNA was stained with Hoechst 33342 (blue). Scale bar, 50 μm. **e** The proportion of beating cardiomyocyte-like cells induced from fPSCs and mESCs (EBs differentiation system). fPSCs-1#/2# represented two fPSCs lines (CMTI-1-fPSCs and 46C-fPSCs). All experiments were repeated at least three times. Error bars represented SEM. **f** The beating cardiomyocyte-like cells induced from fPSCs were stained with specific antibody for Ki-67 (proliferation marker), MLC(2V), α-actinin, cTnT and Gata4 (cardiomyocyte markers). Nucleus was revealed by Hoechst 33342 staining. Scale bars, 20 μm. **g** Time-lapse recording of Ca^2+^ releasing from the cardiomyocyte-like cells differentiated from fPSCs after 7–8 day differentiation. Scale bar, 40 μm. **h** Typical traces of simultaneous action potentials (APs) (left panel) and typical ventricle-like (middle panel) and atrium-like APs (right panel) observed in the cardiomyocyte-like cells differentiated from fPSCs after 7–8 days differentiation.

Next, we investigated the differentiation efficiency of fPSCs towards three germ layers in vitro. For neuroectoderm differentiation, *Sox1-GFP* reporter fPSCs were derived from 46C mESCs, and were negative for GFP initially (Supplementary information, Fig. S8a). *Sox1-GFP* fPSCs were induced in neural differentiation conditions and GFP-positive cells were analyzed by flow cytometry analysis as we previously reported.^34,64^ After differentiation of fPSCs, Sox1-GFP-positive cells rapidly appeared at day 1 (> 20%) and at day 1.5 (> 80%) (Supplementary information, Fig. S8b). The efficiency of fPSC differentiation into Sox1-GFP^+^ cells was ~94% at day 2, while the efficiency of EpiLCs was ~73% at day 2 and the efficiency of mESCs was 68% in monolayer induction system after 6.5 days of differentiation (Fig. 6a; Supplementary information, Fig. S8b). After 4 days of neural differentiation, fPSCs formed typical neural rosette structures, as shown by the staining of tight junction protein 1 (ZO-1) and the neural stem cell marker Nestin (Fig. 6b). Tuj1^+^ neurons were observed by day 7 (Fig. 6b).

To investigate the potential of endodermal differentiation, fPSCs were induced into hepatocyte-like cells as previously reported (Supplementary information, Fig. S8a).^65^ By withdrawing XAV939 from the medium, the migration of some cells was observed. At day 5 after differentiation, the cells were stained with the endoderm marker Foxa2. The differentiation of fPSCs into Foxa2^+^ endoderm cells was at an efficient of 88% at day 5 (Fig. 6c). Next, the cells were cultured for an additional 7 days of differentiation and stained with the specific antibody for AFP (Alpha-fetoprotein, a liver cell marker). Our results showed that the cells were positive for AFP (Fig. 6d).

To investigate the potential of mesodermal differentiation, fPSCs were induced into cardiomyocyte-like cells as described previously (Supplementary information, Fig. S8a).^66^ Beating cardiomyocyte-like cells were observed in most fPSC colonies after 4 days of differentiation (Supplementary information, Video S2). When beating fPSC-derived colonies were counted, the efficiency of fPSC differentiation was ~90% (Fig. 6e). Cardiomyocyte-like cells differentiated from fPSCs expressed the proliferative marker Ki-67 and the cardiomyocyte-specific markers MLC(2V), α-actinin, cTnT and Gata4 (Fig. 6f). Well-organized sarcomere myofilaments with cross-striations were observed in the cardiomyocyte-like cells (Fig. 6f). These cardiomyocyte-like cells were further matured for an additional 3–4 days and examined for their Ca^2^^+^ release and electro-physiology, the classical characteristics of cardiomyocytes. Time-lapse microscopy recorded typical calcium transient signals in these cardiomyocyte-like cells (Fig. 6g; Supplementary information, Video S3), indicating their functional calcium handling. Electrophysiological recordings revealed cardiomyocyte-like cells with forceful atrial- and ventricular-like action potentials (Fig. 6h).

RSCs, EpiLCs and cryopreserved fPSCs were also differentiated in vitro as described above (Supplementary information, Fig. S8a). The differentiation kinetics of RSCs into neural progenitors and PGCLCs were similar to naïve mESCs, but different from fPSCs (Supplementary information, Fig. S8b–d). Beating cardiomyocyte-like colonies were not observed in the differentiated EpiLCs even after 12 days of induction (Supplementary information, Fig. S8e). Cryopreservation did not affect the differentiation of fPSCs into neural progenitors and cardiomyocyte-like cells (Supplementary information, Fig. S9a–e; 94% Sox1-GFP^+^ and ~90% Beating colonies). Together, these results suggest that fPSCs have high potency for rapid differentiation into the three germ layers in vitro.

### fPSCs contribute to three germ layers in vivo

**Fig. 7:**
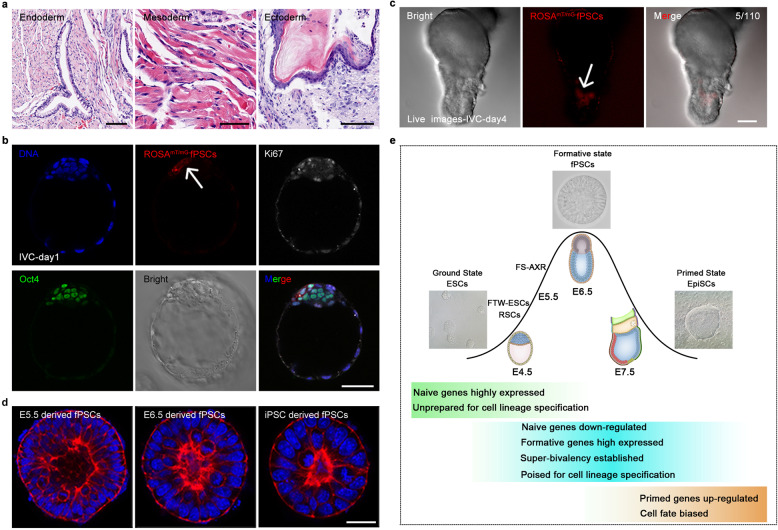
fPSC engraftment in vivo and derivation from in vivo epiblasts. **a** Teratomas generated in vivo from fPSCs (passage 6) were fixed and sliced for paraffin sections. The paraffin sections were subjected to H&E staining. Left panel, endodermal gland-like structure; middle panel, mesodermal sarcomere-like structure; right panel, keratinized epidermis-like cells. Scale bar, 100, 50, 100 μm, respectively. **b** *ROSA*^*mT/mG*^-fPSCs were injected into 134 blastocysts (seven independent experiments) and cultured for 24 h. 25 embryos had tdTomato^+^ cells (red) in the ICM. The injected embryos were stained with specific antibodies for Ki67 (white) and Oct4 (green). DNA was labeled with Hoechst 33342 (blue). Arrows pointed the descendant of *ROSA*^*mT/mG*^-fPSCs (red) in the ICM. Scale bar, 50 μm. **c** The 109 blastocysts (five independent experiments from **b**) injected with *ROSA*^*mT/mG*^-fPSCs were cultured for 4 days in vitro. The images were captured for the injected embryos with fluorescent confocal microscopy. Arrows indicate the descendants from *ROSA*^*mT/mG*^-fPSCs tdTomato^+^ cells (red) in epiblasts of embryos (*n* = 5). Scale bar, 50 μm. **d** fPSCs were established from mouse E5.5, E6.5 epiblasts, and iPSCs. The apical lumens of fPSCs were labeled by Phalloidin (red) staining. DNA was revealed by Hoechst 33342 (blue) staining. Scale bar, 20 μm. **e** A model and characterizations of pluripotent states of embryonic stem cells and epiblasts in early mouse embryos.

For in vivo differentiation, we subcutaneously injected two cell lines of fPSCs (CMTI-1 and *ROSA*^*mT/mG*^) into SCID (severe combined immunodeficient) mice (*n* = 8). After 4–6 weeks, the teratomas were collected for hematoxylin and eosin (H&E) staining (Supplementary information, Fig. S10a). The results showed that the teratoma generated from fPSCs was composed of three germ layer cells: endoderm (endotheliocytes, ciliated endodermal epithelia), mesoderm (muscle cells, adipocytes, erythrocytes) and ectoderm cells (keratinized epidermal cells) (Fig. 7a; Supplementary information, Fig. S10b). Three germ layer derivatives were also observed in the teratoma by immunofluorescent staining with three germ layer markers, Foxa2 for endoderm, α-actinin and α-SMA for mesoderm, Tuj1 and GFAP for ectoderm (Supplementary information, Fig. S10c).

For in vivo blastocyst chimerism assays, injected *ROSA*^*mT/mG*^-fPSCs were observed to contribute to the ICM of 18.7% embryos (25/134) at 24 h post-injection (Fig. 7b; Supplementary information, Fig. S10d), while *ROSA*^*mT/mG*^-mESCs integrated into the ICM of 100% embryos (36/36). The incorporated fPSCs were positive for Ki67 and Oct4, but negative for the trophectoderm marker Cdx2 (Fig. 7b; Supplementary information, Fig. S10e). When the injected embryos were cultured for 4 days and allowed to develop into egg cylinders in vitro,^67^ the tdTomato^+^ cell progeny of fPSCs were observed in 4.5% of mouse embryos (5 out of 110) (Fig. 7c), while *ROSA*^*mT/mG*^-mESCs incorporated into the egg cylinders of ~92% embryos (33/36). Although ~18% chimeric pups (3/17) were born from the injection of *ROSA*^*mT/mG*^-mESCs, no chimeras were found among 181 pups, as judged by coat and eye color, after 387 blastocysts were injected with fPSCs and transferred into pseudopregnant females (two cell lines: CMTI-1, 0/168; *ROSA*^*mT/mG*^-mESCs, 0/219). Altogether, these results suggest fPSCs possess pluripotency according to teratoma and blastocyst chimerism assays, but the chimerized cells show only limited survival in postimplatation embryos, suggesting that the assay for chimerism with pre-implantation blastocysts might not be suitable for fPSCs, which may represent early-gastrulation epiblast cells.

### Stabilized fPSCs derived from pre-/early-gastrula epiblasts or iPSCs

Stablized fPSCs could be efficiently established from intact epiblasts at E5.5 (*n* = 7/7, success number/total epiblast number) or E6.5 (*n* = 8/8) mouse embryos, as well as iPSCs (Fig. 7d). Stabilized fPSCs could also be efficiently derived from the fragments of E6.5 epiblasts (PPr, posterior-proximal fragments, *n* = 3/8; PD, posterior-distal fragments, *n* = 5/10; APr, anterior-proximal fragments, *n* = 14/15; AD, anterior-distal fragments, *n* = 2/2). However, fPSCs were difficult to establish from late-gastrulation E7.5 epiblasts and no fPSCs were obtained from VE (data not shown). fPSCs derived from epiblasts expressed pluripotency markers (Oct4, Sox2 and Nanog) (Supplementary information, Fig. S10f). Similar to the fPSCs derived from mESCs, these fPSCs had high capacity for differentiation into cardiomyocyte-like cells and PGCLCs (Supplementary information, Fig. S10g, h; 89.6% beating colonies, 12.5% SSEA1^+^/CD61^+^ cells). RNA-seq analysis suggested that the epiblast derived fPSCs had similar transcriptomes with those induced from mESCs (Fig. 4a; Supplementary information, Fig. S4a). The expression levels of key genes in the fPSCs derived from epiblasts were similar to those in the fPSCs induced from mESCs (Supplementary information, Fig. S10i).

## Discussion

Pluripotent stem cells can undergo long-term self-renewal in vitro, but pluripotency is a transient state in vivo, lasting only a few days between the pre-implantation blastocyst and the post-implantation gastrula, thus making it a difficult topic to study. Substantial progress has been made in the understanding of epiblast pluripotency and mammalian early embryonic development ever since ESCs and EpiSCs were established.^9–12^ These different types of pluripotent stem cells led to the model that pluripotency can be conceptualized as a dichotomy between naïve and primed pluripotency, and that the naïve state could be connected to embryonic diapause.^20,68^ However, subsequent studies suggested that a model with just these two pluripotent states could be over-simplified, and it fails to recapitulate the dynamically changing potencies of the epiblast during early postimplantation development in mammals. Thus, multiple states of formative pluripotency were proposed,^23–25^ however conclusive evidence of their existence had remained elusive.

At present, the derivation of stable and self-renewing fPSCs supports the existence of formative pluripotency states between naïve ESCs and primed EpiSCs. Firstly, fPSCs can be derived from epiblasts (E5.5, E6.5), mESCs (CMTI-1, 46C, *BVSC* and *ROSA*^*mT/mG*^) and iPSCs through the inhibition of EMT and mimicking the in vivo environment of developing embryos, demonstrating the feasibility, stability and accessibility of this pluripotent state in vitro. Secondly, similar to the epiblasts but unlike mESCs and EpiSCs, fPSCs express high levels of formative pluripotency markers and low levels of naïve and lineage-specific markers. Thirdly, in terms of the epigenome, fPSCs is most similar to the E6.5 epiblasts, not mESCs nor EpiSCs. Most importantly, even after long-term propagation, fPSCs retain the capacity to differentiate into PGCs, as well as the three germ layers, probably similar to the pre-/early-gastrula epiblasts. In addition, several stable cells of intermediate PSCs (RSCs, FTW-ESCs and FS-AXR) were most recently reported between naïve and primed state, further supporting the existence of formative pluripotency in mammalian early development. Although these intermediate PSCs are similar in many formative features, fPSCs are derived in 3D conditions and hold unique epigenetic states that is similar to E6.5 epiblasts, suggesting that fPSCs are different from RSCs, FTW-ESCs and FS-AXR (Fig. 7e and Table 1). Altogether, our results suggest that fPSCs are a novel stem cell line that captures a formative pluripotent state between naïve and primed pluripotency, probably representing the early-gastrula epiblast cells.

**Table 1:** Features of mouse pluripotent stem cells.

Feature	mESCs	RSCs	FTW-ESCs	FS cells	fPSCs	EpiSCs
Pluripotency	Naïve	Intermediate	Formative	Formative	Formative	Primed
Colony morphology	Domed	Flat/Rossette	Domed/Rosette	Flat	Rosette	Flat
Embryonic equivalent	E3.75–4.5 ICM	E5.0 epiblasts	E5.0–6.0 epiblasts	E5.5–6.0 epiblasts	E6.0–6.5 epiblasts	E7.5 anterior primitive streak
Polarity	No	Yes	Yes	Yes	Yes	Yes
Naïve TF expression level	High	High	Medium	Low	Low	Low
Formative TF expression level	Low/middle	Low/Medium	High/Medium	High	High	Medium/Low
Lineage-biased TF expression level	Low	Low	Medium/Low	Low	Low	High
Growth factors requirement	Lif and Wnt on, MEK off	Lif on, Wnt and MEK off	Fgf on, TGF-β on, Wnt on	Activin A on, Wnt and RA off	Activin A and MEK on, Wnt off	Activin A and MEK on, Wnt off
Culture environment	2D	2D	2D	2D	3D	2D
Single cell propagation	Yes	Yes	Yes	Low (without ROCKi)	Yes	No (without ROCKi)
Formation of blastocyst chimaeras	Yes	Yes	Yes	Yes	Partial	No*
X inactivation	XaXa	XaXa	XaXa	XaXi	XaXi	XaXi
DNA methylation level	Low	Medium	No data	No data	High	High
Super-bivalency	Medium	Medium	Medium	Medium	High	Medium
Responsiveness to differentiation	Slow	Slow	Rapid	Rapid	Rapid	Fate pre-determined
Germ cell competence (in vitro)	Barely**	Barely**	Yes	Yes	Yes	Barely***

*TF* transcription factor, No* Except Oct4-GFP^+^ subpopulation, Barely** Unless EpiLCs induced, Barely*** very low.

After embryos implant into the uterus, epiblast cells undergo a maturation process with dramatic transformation in their morphology, transcriptome, epigenome, signaling pathways and metabolism, in preparation for morphogenetic patterning and lineage specifications following gastrulation.^23,24^ Due to a myriad of technical difficulties associated with inaccessibility, the process of epiblast development remains a mystery in mammals. The multiple PSCs between naïve and primed state will provide good opportunities for the investigations of molecular and epigenomic regulation events during mammalian early postimplantation development. For example, although 2D-derived mouse EpiLCs have transcriptomic profiles similar to fPSCs, they have limited capacities for differentiation into the three germ layers, probably consistent with their aberrantly high DNA methylation at the promoters of lineage specification markers. In comparison, mouse EpiSCs barely differentiate into PGCLCs in vitro, probably consistent with their high DNA methylation of the DMV of *Oct4*, whose expression is tightly regulated during gametogenesis.^69^ All these lines of evidence suggest that 3D culture conditions coupled with Wnt inhibition to prevent EMT, may provide a more suitable environment to maintain the epigenetic identity and thus the differentiation potential of mouse early-gastrula epiblast cells in vitro. Thus, the fPSCs established in this study provide an unique opportunity to investigate the possible role of epigenetic factors in the development of mouse epiblast cells. Deep investigations of fPSCs and other intermediate PSCs will likely further improve our understanding of mammalian early postimplantation development.

Human ESCs hold enormous hope for regenerative medicine and tremendous efforts have been made to differentiate these cells into various types of functional cells in the body, including PGCs and gametes. However, the heterogeneity, variability and inefficiency of directed differentiation in vitro has impeded the clinical applications of these cells.^13^ The establishment of fPSCs by mimicking mammalian embryonic development in vivo may contribute to solve these issues. Further investigation of fPSCs in mammals could potentially improve directed differentiation of ESCs and iPSCs in general, with important implications for the translational and clinical applications of PSCs in the future.

## Materials and methods

### Animal treatment and ethic statements

Animal maintenance and experiments were performed in line with the guidelines of the Institutional Animal Welfare and Use Committee at Institute of Zoology, Chinese Academy of Sciences. SCID (severe combined immunodeficient) and normal CD1 mice were purchased from Charles River Laboratories (Beijing).

### Culture of mESCs, iPSC and EpiSCs

mESCs, iPSCs and EpiSCs were generated as previously reported.^34,37,70^ mESCs and iPSCs were cultured on feeder-free dishes coated with 0.2% gelatin (Millipore, Cat# 901771) in the N2B27^2i/lif^ medium, which included N2B27 medium supplemented with 1 μM PD035901 (LC Laboratories, Cat# P-9688), 3 μM Chir99021 (LC Laboratories, Cat# C-6556), and 1 × 10^3^ units/mL hLIF (human Leukemia Inhibitory Factor, Millipore, Cat# ESG1107). EpiSCs were cultured on mouse MEFs (Embryonic Fibroblast cells) inactivated with Mitomycin C (Sigma-Aldrich, Cat# M0503) in N2B27 medium containing 15% KSR (Gibco, Cat# 10828028), 20 ng/mL Activin A (PeproTech, Cat# 100-18B), 12 ng/mL bFGF2 (R&D Systems, Cat# 233-FB) and 5 μM XAV939 (Sigma-Aldrich, Cat# X3004). EpiSCs were passaged every 2–3 days as small clumps with 1.5 mg/mL collagenase IV (Gibco, Cat# 17104-019) or as single cells with TrypLE (Gibco, Cat# 12605010) digestion through adding Y-27632 (TOCRIS, Cat# 1254).

### Establishment and maintenance of fPSCs

For the derivation of Epiblastoids, mESCs were washed once with DPBS (Sigma-Aldrich, Cat# D5652), incubated at 37 °C with 0.25% Trypsin-EDTA (Invitrogen, Cat# 25200072) for 1 min, and added with equal volume of medium containing FBS (fetal bovine serum, Millipore, Cat# ES-009-B) to terminate the digestion. The clones were pipetted into single cells, collected by centrifugation (1000× *g*/min, 3 min), and washed twice with N2B27 medium. Then, the single cells were suspended and added with equal volume of Matrigel (BD, Cat# BD356230). The cells were plated and incubated at 37 °C for 1 h until Matrigel solidification. The cells embedded in Matrigel were filled with pre-warmed N2B27 medium supplemented with KSR, Activin A and bFgf2, and cultured at 37 °C and 5% CO_2_ until analysis.

To maintain Epiblastoids, XAV939, SB431542 (Axon, Cat# 1661) and Verteporfin (Selleck Chemicals, Cat# S1786) were added to the culture medium supplemented with KSR, Activin A and bFgf2. The Epiblastoids were finally stabilized by the treatment with XAV939. Thus the finalized medium for stabilized Epiblastoids (fPSCs) was N2B27 medium supplemented with Activin A, bFgf2, and XAV939.

For the propagation of fPSCs, colonies were suspended with pre-cold DPBS, washed with DPBS for one time. The cells were treated with TrypLE for 1 min at 37 °C to dissociate into single cells and were collected by centrifugation (1000× *g*/min, 3 min). These cells were re-suspended in fPSC medium and added with equal volume of Matrigel. The cells with Matrigel were plated and incubated at 37 °C for 1 h. After Matrigel solidification, the cells were added with additional fPSC medium, and cultured at 37 °C and 5% CO_2_. fPSCs were propagated every 2.5–3 days. The cryopreservation medium for fPSCs contained 90% FBS and 10% DMSO (dimethylsulfoxide, Sigma-Aldrich, Cat# D8418).

Mouse early postimplantation embryos were isolated from the timed pregnant females. After digestion of the embryonic tissues with PT (Protease and Trypsin) for 3 min at 4 °C, visceral endoderm was removed through mouth pipetting under microscope. The embryonic fragments of epiblast and iPSCs were embedded in Matrigel and fPSC medium and cultured in fPSC medium for 3–4 days.

### Immunostaining analysis

The cells, whole-mount blastocysts and whole-mount embryos at egg-cylinder stage were fixed with 4% paraformaldehyde (PFA) (Sigma-Aldrich, Cat# 158127) for 15 min at room temperature. For paraffin section staining, the antigens were retrieved by microwave in the solution of sodium citrate after the samples were dewaxed and rehydrated according the standard processes. After washing 3–5 times with DPBS containing 0.1% Triton X-100 (Amresco, Cat# 0694), the samples were penetrated with DPBS containing 1% Triton X-100 for 15 min and blocked with 0.5% BSA (Sigma-Aldrich, Cat# A4378) in DPBS at room temperature for 1 h. Then the samples were incubated with the primary antibodies overnight at 4 °C. After washing three times with DPBS, the samples were incubated 1 h with the second antibodies at room temperature. DNA was stained with Hoechst 33342 (Invitrogen, Cat# H3570). Images were acquired with Zeiss microscope (LSM 780; Carl Zeiss).

The primary antibodies included: rabbit anti-Ezrin (Abcam, Cat# Ab76247, 1:200); mouse anti-GM130 (BD biosciences, Cat# BD610822, 1:200), rat anti-Nestin (Millipore, Cat# MAB353, 1:50–1:100); rabbit anti-Zo-1 (Abcam, Cat# Ab214228, 1:150), rabbit anti-Tuj1 (Sigma-Aldrich, Cat# T2200, 1:100), goat anti-Oct4 (Santa Cruz Biotechnology; Cat# sc-8628, 1:200), rabbit anti-Nanog (Abcam; Cat# ab80892, 1:200), mouse anti-Sox2 (CST; Cat# 4900S, 1:200), rabbit anti-Ki-67 (Abcam, Cat# ab15580, 1:500), mouse anti-cardiac troponin T (cTnT, Thermo Fisher Scientific, Cat# MS-295-P0, 1:400), mouse anti-α-actinin (Sigma-Aldrich, Cat# A7732, 1:500), rabbit anti-myosin light chain (MLC) 2v (ProteinTech Group, Cat# 10906-1-AP, 1:50), rabbit anti-Foxa2 (CST, Cat# 8186S, 1:200), goat anti-Gata6 (R&D systems, Cat# AF1700, 1:200), goat anti-Otx2 (R&D systems, Cat# AF1979, 1:100), goat anti-Gata4 (Santa Cruz Biotechnology, Cat# sc-1237, 1:200), rabbit anti-E-Cadherin (Santa Cruz Biotechnology, Cat# sc-7870, 1:200), mouse anti-α-SMA (Santa Cruz Biotechnology, Cat# sc-53142, 1:200), mouse anti-GFAP (Sigma-Aldrich, Cat# G3893, 1:100), mouse anti-AFP (CST, Cat# 3903S). The secondary antibodies were from Jackson ImmunoReseach included: Alexa Fluor® 488 AffiniPure Donkey Anti-Rabbit IgG (H + L) (Cat# 711-545-152, 1:200); Alexa Fluor® 594 AffiniPure Donkey Anti-Rabbit IgG (H + L) (Cat# 711-585-152, 1:200); Cy™5 AffiniPure Donkey Anti-Rabbit IgG (H + L) (Cat# 711-175-152, 1:200); Alexa Fluor® 488 AffiniPure Donkey Anti-Mouse IgG (H + L) (Cat# 711-545-150, 1:200); Alexa Fluor® 647 AffiniPure Donkey Anti-Mouse IgG (H + L) (Cat# 715-605-151, 1:200); Alexa Fluor® 594 AffiniPure Donkey Anti-Mouse IgG (H + L) (Cat# 715-585-150, 1:200); Alexa Fluor® 488 AffiniPure Donkey Anti-Goat IgG (H + L) (Cat# 705-545-003, 1:200); Alexa Fluor® 488-AffiniPure Donkey Anti-Rat IgG (H + L) (Cat# 712-545-153, 1:200).

### Time-lapse recording and fluorescence activated cell sorting

For time-lapse experiments, *ROSA*^*mT/mG*^ mESCs were derived from *ROSA*^*mT/mG*^ mice that were purchased from the Nanjing Biomedical Research Institute of Nanjing University as described previously.^34^ After culture of 3 days, time-lapse imaging of cell migration from the colonies was performed by using the Ultra VIEW-Vox confocal imaging system (PerkinElmer). The images were captured every 15 min for 24 h. The data were processed with the software of Velocity software 6.0 (PerkinElmer).

For the flow cytometry analysis, the differentiated ESCs or fPSCs colonies with reporters were washed with DPBS, incubated with TrypLE for 1 min at 37 °C for digestion, added with FBS to terminate the digestion. The cells were dissociated into single cells by pipetting and were collected by centrifugation at 1000× *g*/min for 3 min. After washing with DPBS, the cells were filtered with a cell strainer cap (Falcon™ Cell Strainers, Cat# 352235) and immediately analyzed by flow cytometry (BECKMAN COULTER Life Sciences). Mouse *Sox1-GFP* ESCs (46C) were used for the analysis of the formation of neural precursor as we described previously.^34^ For the analysis of PGCLCs induction from fPSCs, PGCLC aggregations were dissociated into single cells by trypsin-EDTA for 7–8 min at 37 °C and pipetted every 3 min during this process. Then, FBS was added to terminate the digestion reaction. The cells were stained with PE anti-CD61 (BioLegend, Cat# 104307, 1:200) and anti-SSEA1 Alexa Fluor 647 (eBioscience, Cat# 51-8813, 1:20) for flow cytometry analysis. Data analysis was performed with FlowJo 7.6 software (Beckman Coulter MoFlo XDP).

### Quantitative RT-PCR

mRNA was isolated from the cells and embryonic fragments of epiblast with RNAzol (Mrcgene, Cat# RN190) according to the protocol of manufacturer. The concentration of mRNA was measured by NanoDrop 2000 (Thermo Fisher Scientific). For each sample, 500 ng mRNA was transcribed into cDNA with PrimeScript RT Reagent Kit (TaKaRa, Cat# RR037A). Quantitative PCR (qPCR) was performed with EvaGreen 2× qPCR MasterMix (ABM, MasterMix-S) on LightCycler 480 II (Roche). All results were repeated with at least three independent samples. *Gadph* or β*-actin* was used to normalize the gene expression. The 2^−ΔCt^ method was used for data analysis.^71^ All primer sequences for qPCR were listed in Supplementary information, Table S6.

### Western blot

The samples were separated by SDS-PAGE and transferred into PVDF membrane. The membrane was incubated with the primary antibodies at 4 °C overnight. After washing with PBS, the secondary antibodies (Jackson ImmunoResearch) were added and incubated at room temperature for 1 h. The signal was developed with SuperSignalTM West Femto Maximum Sensitivity Substrate (Thermo Fisher Scientific, Cat# 34095) and was observed with BIO-RAD ChemiDocTMXRS + (Bio-Rad). The results were processed with Quantity One software (Bio-Rad).

The following primary antibodies were used: mouse anti-β-actin (Yeasen, Cat# 30101ES50, 1:2000), goat anti-Oct4 (Santa Cruz Biotechnology, Cat# sc-8628, 1:1000), mouse anti-Sox2 (CST, Cat# 4900S, 1:1000), rabbit anti-Nanog (Abcam, Cat# ab80892, 1:1000), goat anti-T (CST, Cat# 81694, 1:1000), goat anti-Otx2 (R&D systems, Cat# AF1979, 1:1000), rabbit anti-Foxa2 (CST, Cat# 8186S, 1:200); rabbit anti-E-Cadherin (Santa Cruz Biotechnology, Cat# sc-7870, 1:1000), mouse anti-N-Cadherin (BD, Cat# 610920, 1:1000). The secondary antibodies were from Jackson ImmunoResearch, including Peroxidase AffiniPure Goat Anti-Rabbit IgG (Cat# 111-035-003, 1:3000), Peroxidase AffiniPure Goat Anti-Mouse IgG (Cat# 115-035-003, 1:3000), Peroxidase AffiniPure Rabbit Anti- Goat IgG (Cat# 305-035-003, 1:3000).

### Alkaline phosphatase staining

Alkaline phosphatase staining was performed with BCIP/NBT Alkaline Phosphatase Color Development Kit (Beyotime, Cat# C3206). Briefly, cells were washed three times using PBS and fixed with 4% PFA for 30 s at room temperature. After washing three times with PBST, the cells were stained by BCIP/NBT solution for 30 min at room temperature. The images were obtained with Zeiss LSM 780 microscope (Carl Zeiss).

### Cell growth and cloning efficiency analysis of PSCs

Cells were cultured and digested into single cells. The number of the cells was counted with hemocymeter. The cells were re-plated on 35 mm dish at a density of 3 × 10^5^/mL (30,000 cells per well) in triplicates, and cultured in specific medium at 37 °C and 5% CO_2_. The clones were counted at day 1, 2, 3, respectively. The data were calculated as the exponential growth curve.

For the analysis of cloning efficiency, the counted single cells of PSCs were plated on 24-well plate (1000 cells per well) in triplicates. These cells were cultured in specific medium with or without Y27632, N2B272i/lif medium and EpiSCs medium supplemented with Y27632. After culture of 3 days, the cells were counted and the cloning efficiency.

### Karyotype analysis

Cells were cultured for 3 days and added with colchicine (0.02 mg/mL) to block the cell cycle progression for 1 h. The metaphase cells were dissociated and collected after the washing three times with PBS. These cells were suspended and incubated with hypotonic KCl solution (0.56%) for 6 min to obtain single cell suspension. Then, the cells were fixed 4 times with 5 mL ice-cold fixative solution (methanol:acetic acid = 3:1) and collected through centrifugation. Cell pellets were re-suspended with 200 μL fixative solution, spread onto the ice-cold slides, and stained with Giemsa solution for 2 h at room temperature. After washing three times with PBS, the chromosome spread was detected by mercury lamp and analyzed by Videotest software.

### Induction of PGCLCs and three germ layers

Mouse EpiLCs and PGCLCs induction were performed as previously reported.^21^ Briefly, EpiLCs or fPSCs were dissociated to single cells with TrypLE for 1 min at 37 °C. And the cells were plated in a well of low cell attachment U-bottom 96-well plate (Corning, Cat# 7007) in G-MEM medium (1×) (Gibco, Cat# 11710-035) supplemented with 15% KSR, 1 mM sodium pyruvate (Gibco, Cat# 11360070), 0.1 mM non-essential amino acids (Gibco, Cat#11140050), 0.1 mM b-mercaptoethanol (Invitrogen, Cat# 21985-023), 2 mM L-glutamine (Gibco, Cat# 35050061) and 100 units/mL penicillin, 0.1 mg/mL streptomycin (Gibco, Cat# 15140122) (GK15 medium), at the presence of the growth factors, including 500 ng/mL BMP4 (R&D Systems, Cat# 315-27), 100 ng/mL SCF (R&D Systems, Cat# 455-MC-010), 500 ng/mL BMP8a (R&D Systems, Cat# 1073-BP-010), 50 ng/mL EGF (R&D Systems, Cat# 2028-EG), and 1000 units/mL hLIF.

Neural precursor induction of mESCs was performed with monolayer differentiation as previously reported (Supplementary information, Fig. S6a).^34,64^ Firstly, fPSCs were derived from *Sox1-GFP* reporter mESCs (46C). Then the clones were suspended and washed twice with pre-cold DPBS, seeded on ultra-low attachment dishes, and cultured with N2B27 medium containing 6 μM PD0325901 for the first day. The culture medium was replaced with fresh N2B27 basal medium for the following culture. After the majority of cells were Sox1-GFP positive at day 2, the culture medium was changed every day and the cells were stained with specific antibodies at specific time points.

For cardiomyocyte generation, the protocol was optimized from previous reports (Supplementary information, Fig. S6a).^66^ After day 3 culture, the medium was changed with 20 ng/mL Activin A and 12 ng/mL bFgf2 for the differentiation of fPSCs. One day later, the medium was changed with RPMI medium 1640 (1×) (Gibco, Cat# 11875-093) supplemented with 4 mM VC (Vitamin C, Sigma-Aldrich, Cat# A7506) and B27 supplement minus insulin (Gibco, Cat# A1895601), termed Basal Medium 1 (BM1) for the culture of additional one day. Then, 10 μM XAV939 was added into the medium. The cells were cultured in these conditions for another day and the medium was changed to BM1. After the emergence of beating clones, the medium was refreshed everyday with RPMI 1640 supplemented with 4 mM VC and normal B27 supplement plus insulin, termed Basal Medium 2 (BM2). The cardiomyocyte differentiation through EB formation was performed as previously reported.^72^

Hepatic-like cell induction was performed as previously reported (Supplementary information, Fig. 6a).^65^ Briefly, after fPSCs were grown for 3 days, the medium was changed to the N2B27 medium with 20 ng/mL Activin A and 12 ng/mL bFgf2; and the cells were cultured in these conditions for 3–5 days at 37 °C, 5% CO_2_ and 20% O_2_ (normal oxygen concentration). Then the medium was changed to the medium containing RPMI 1640, B27 supplement minus insulin and Activin A (100 ng/mL) for the culture of additional two days. Next, the medium was changed to RPMI1640, B27 supplement, 10 ng/mL Fgf2 and 20 ng/mL BMP4; the cells were cultured for the subsequently 2 days at 37 °C, 5% CO_2_ and 4% O_2_ (normal oxygen concentration). Then the medium was changed to RPMI 1640 containing B27 supplement, 2.5% FBS, 100 nM Dexamethasone (Sigma-Aldrich, Cat# D4902), 10 ng/mL HGF (PeproTech, Cat# 100-39) and 20 ng/mL Oncostatin M (R&D systems, Cat# 295-OM) for the culture of following 5 days at 37 °C, 5% CO_2_ and 4% O_2_. The cells were stained with the antibodies at specific time points.

### Electrophysiology and intracellular Ca^2+^ measurements

The cardiomyocyte-like cells were differentiated from fPSCs and matured for 6 days. The beating colonies (growing on glass cover-slips) were transferred to the patch clamp recording buffer. The electrophysiological experiments were executed by EPC-10 amplifier (Heka, Germany) and the action potential activity was recorded by glass microelectrodes.

For intracellular Ca^2^^+^ measurements, the contracting cardiomyocytes were labeled with Fluo-3 AM (Beyotime, Cat# S1056, 1:5000) in fresh BM2 at 37 °C and 5% CO_2_ for 1 h. The cells were washed twice with fresh BM2, and then were added with 2 mL fresh BM2. The Fluo-3 AM fluorescence of Ca^2^^+^ in the cells was recorded by using Ultra VIEW-Vox confocal imaging system at 488 nm excitation and emission (PerkinElmer).

### Teratoma formation

fPSCs were dissociated into single cells and suspended in serum-free DMEM basic medium at a destiny of 2 × 10^6^ cells/100 μL. The single cells were injected subcutaneously into groin of 6-weeks-old SCID males. After 4–6 weeks, the teratomas were isolated and fixed overnight at 4 °C with 4% PFA. The samples were embedded in paraffin and sliced into 4–5 μm sections. H&E staining of the sections of teratomas were performed according to a standard protocol. The immunofluorescence staining of teratomas was performed as described above. The images of H&E staining were acquired with Leica Aperio VESA8.

### Chimeric assay of fPSCs and culture of postimplantation embryos

fPSCs and mESCs were digested into single cells. The single cells were suspended with M2 medium (Sigma-Aldrich, Cat# M7167) at a density of 10^5^ cells/mL and was filtered with a cell strainer cap. After placing on ice for 20 min, 8–10 cells were injected into the blastocoel cavity of E3.5 blastocysts. 10–15 injected embryos were transferred to the uterine horns of 2.5 d.p.c. (day post-coitum) pseudo-pregnant female mouse. Alternatively, the injected embryos were cultured in vitro as our previous report.^67^ Briefly, the injected blastocysts were cultured the petri dishes coated Matrigel (BD, Cat# 356234) in CMRL 1066 medium (Gibco, Cat# 11530037) supplemented with 10% FBS (Millipore, ES 009B), 100 units of penicillin streptomycin (Invitrogen Cat# 10378016), 1 mM sodium pyruvate (Invitrogen, Cat# 11360070), 2 mM L-Glutamine (Invitrogen Cat# 25030081), N2 (Invitrogen, Cat# 17502048) and B27 supplement (Invitrogen, Cat# 17504044) for first 2 days. Then, the medium was changed every day with the increase of FBS, KSR or homemade rat serum until the embryos were analyzed.

### RNA sequencing and data analysis

Total RNA was isolated from naïve mouse ESCs, d2, d3, d4 Epiblastoids, EpiLCs, EpiSCs, different passages of fPSCs (P1, P10, P20, P30) and fPSCs established from E5.5/6.5 epiblasts by RNA-zol. Genomic DNA was eliminated from the total RNA by the digestion of RQ1 DNase (Promega, Cat# M610A) for 1 h at 37 °C. 3 μg total RNA was used to construct the RNA sequencing libraries. RNA-seq libraries were sequenced on Illumina X-Ten.

Low quality sequencing reads and adapters were removed by cutadapt v1.11,^73^ and then the clean reads were aligned to mouse cDNA database (Ensembl, mm9) using TopHat2 v2.1.1.^74^ FPKM (fragments per kilobase per million) was introduced to calculate gene expression value by Cufflinks v2.1.1.^75^

DEGs were identified by DeSeq2 software,^76^ and the threshold of adjusted *P*-value was 0.05. Heatmaps of differentially expressed genes were further clustered by hierarchical clustering and then were visualized by TreeView software.^77^ 2D PCA analysis was performed and visualized by using all expressed genes (http://www.r-project.org). Dot analysis was used to compare the up- or down-regulated genes in the samples and the Pearson correlation coefficient was used to compare the relevance between different samples. DAVID online analysis (DAVID Bioinformatics Resources 6.8) was used for GO enrichment analysis. The corn plot analysis was performed as we previously reported.^45^

### MethylC-seq library generation and data processing

Genomic DNA was isolated using phenol/chloroform extraction and subsequently ethanol precipitation. Whole-Genome Bisulfite Sequencing was conducted as described previously.^61^ Paired-end DNA sequencing (2 × 150 nucleotides) at Illumina X-Ten platform was used. After trimming low quality sequencing reads and adapters, the clean reads were mapped to mm9 by bs_seeker2 following the default parameters.^78^ Unconverted reads, multiple mapped reads and duplicate reads introduced by PCR were also removed.

### STAR ChIP-seq library generation and data processing

The STAR ChIP was performed as previously described.^79^ Briefly, samples were lysed and digested with MNase. Reaction was stopped by adding 5 μL stop buffer. After adding 45 μL pre-cold 2× RIPA lysis buffer, the solution was spun down, and the supernatant was transferred to a new centrifuge tube containing 40 μL RIPA to remove cell fragments. Then the chromatin samples were incubated with 1 μg antibody for H3K4me3 (in-house) and H3K27me3 (Diagenode, pAb-069-050) overnight with rotation at 4 °C. 100 μg Protein A Dynabeads (Life Technologies) were supplemented to the samples, and incubated with rotation at 4 °C for 2 h. The beads were washed and the supernatant was removed by putting on the magnetic frame. Then, the solution was eluted from the beads and treated with proteinase K. After proteinase K was inactive, the supernatant was transferred to a new tube, 1 μL rSAP (NEB, Cat#: M0371) was employed to dephosphorylate the 3′ end of DNA fragments, and then was inactivated at 65 °C for 20 min. Finally, the samples were subjected to TELP library construction. ChIP-seq libraries were sequenced by Illumina X-Ten following the manufacturer’s protocol.

Sequencing reads were aligned to mouse genome reference (mm9) using Bowtie2 (v. 2.2.2) with the parameters: --t --q --N 1 --L 25 after trimming adapters by Trim Galore (v. 0.4.2).^80^ Multiple mapped reads and PCR duplicates were removed to generate uniquely mapped reads. After validating reproducibility, replicates were integrated for down-stream analysis. The reads per kilobase of transcript per million mapped reads (RPKM) was used to calculate the enrichment of histone modifications at the whole genome level. The UCSC genome browser was used to visualize the signals of STAR ChIP-seq.

### Identification of super bivalent genes

The identification of super bivalent genes was performed as we described previously.^57^ Briefly, the enrichment of H3K4me3 of each sample was calculated within 10-kb promoters and further normalized by *Z*-score. Bivalent genes with H3K4me3 enrichment higher than that of averaged house-keeping genes and strong H3K27me3 in E6.5 epiblast were considered as super bivalent genes. Bivalent genes with H3K4me3 nrichment higher than that of averaged house-keeping genes and strong H3K27me3 with an arbitrary cutoff (normalized RPKM > 1) were considered as super bivalent genes.

### Clustering analysis of DNA methylation and histone modifications

Hierarchical clustering of DNA methylation and histone modification enrichment was conducted with Cluster v.3.0 (uncentered correlation) in 10-kb bins at the whole genome level. Data were visualized by Java TreeView (v.1.1.6r4).

### Single cell analysis

Single cells were obtained from four fPSC clones after digestion of TrypLE at 37 °C for 5 min. Totally, 192 single cells (48 cells/clone) were transferred into the PCR tubes that contained 2.5 μL lysis buffer by mouth pipetting. And then the transcriptome library construction were performed according to the protocol described previously.^67^ The Smart-seq2 data of paired-end reads were processed by the standard pipeline of Drop-seq_tools-2.0.0 (http://mccarrolllab.com).

## Supplementary information

Supplementary Figure S1

Supplementary Figure S2

Supplementary Figure S3

Supplementary Figure S4

Supplementary Figure S5

Supplementary Figure S6

Supplementary Figure S7

Supplementary Figure S8

Supplementary Figure S9

Supplementary Figure S10

Supplementary Table S1

Supplementary Table S2

Supplementary Table S3

Supplementary Table S4

Supplementary Table S5

Supplementary Table S6

Supplementary Video S1 The occurrence of EMT in Epiblastoids beyond 3 Days

Supplementary Video S2 Beating colonies differentiated from fPSCs

Supplementary Video S3 Ca^2+^ release and spark in the cardiomyocyte-like cells

## Data Availability

All data generated in the current study are available in the Gene Expression Omnibus with accession number GSE154290.
