# Author Correction: Formative pluripotent stem cells show features of epiblast cells poised for gastrulation

**DOI:** 10.1038/s41422-022-00695-x

**Published:** 2022-07-12

**Authors:** Xiaoxiao Wang, Yunlong Xiang, Yang Yu, Ran Wang, Yu Zhang, Qianhua Xu, Hao Sun, Zhen-Ao Zhao, Xiangxiang Jiang, Xiaoqing Wang, Xukun Lu, Dandan Qin, Yujun Quan, Jiaqi Zhang, Ng Shyh-Chang, Hongmei Wang, Naihe Jing, Wei Xie, Lei Li

**Affiliations:** 1 State Key Laboratory of Stem Cell and Reproductive Biology, Innovation Academy for Stem Cell and Regeneration, Beijing Institute for Stem Cell and Regenerative Medicine, Institute of Zoology, University of Chinese Academy of Sciences, Chinese Academy of Sciences, 100101 Beijing, China; 2 Department of Cell Biology and Genetics, School of Basic Medical Sciences, Chongqing Medical University, Chongqing, 400016 China; 3 State Key Laboratory of Cell Biology, CAS Center for Excellence in Molecular Cell Science, Shanghai Institute of Biochemistry and Cell Biology, Chinese Academy of Sciences, Shanghai, 200031 China; 4 Center for Stem Cell Biology and Regenerative Medicine, MOE Key Laboratory of Bioinformatics, THU-PKU Center for Life Sciences, School of Life Sciences, Tsinghua University, 100084 Beijing, China; 5 Guangzhou Regenerative Medicine and Health Guangdong Laboratory (GRMH-GDL), Guangzhou Institutes of Biomedicine and Health, Chinese Academy of Sciences, Guangzhou, 510530 Guangdong China

**Keywords:** Embryonic stem cells, Embryonic stem cells

Correction to: *Cell Research* 10.1038/s41422-021-00477-x, published online 19 February 2021

We apologize for the mistake of correspondence email address of Wei Xie. The correct information is as follows. This correction does not affect the conclusion of the work.

Correspondence: Wei Xie (xiewei121@tsinghua.edu.cn) or Lei Li (lil@ioz.ac.cn)

