# Supplementary material for: Formative pluripotent stem cells show features of epiblast cells poised for gastrulation
Source: Cell Res. 2021 Feb 19;31(5):526–41. doi: 10.1038/s41422-021-00477-x (PMC8089102; doi:10.1038/s41422-021-00477-x)
Supplement: Supplementary file 1 — Supplementary Figure S1 [file 41422_2021_477_MOESM1_ESM.pdf]

Supplementary figures and their legends

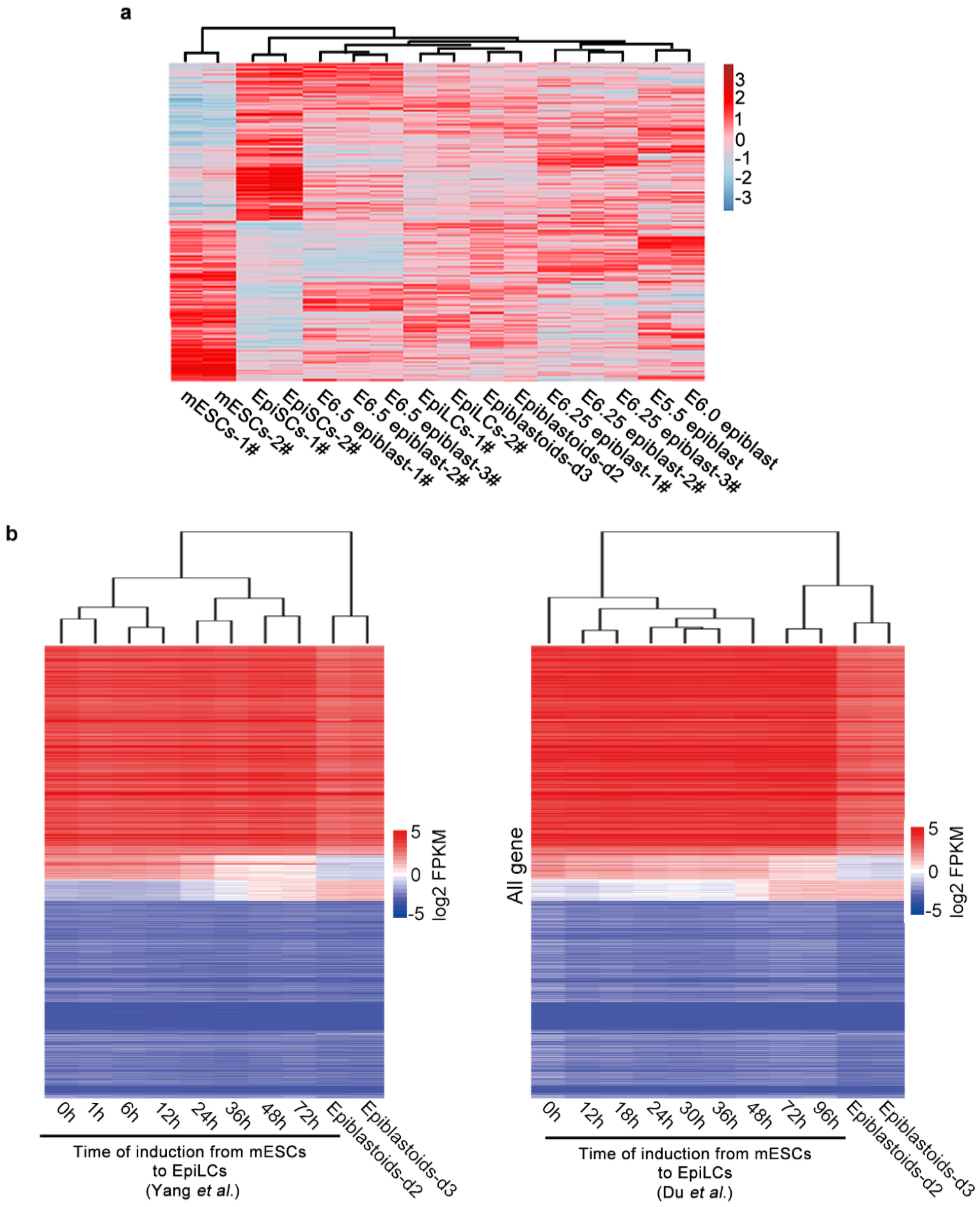

Fig. S1 Hierarchical clustering analysis of RNA sequencing.

a Heatmap of the differentially expressed genes (DEG) among mESCs, EpiLCs, Epiblastoids at day-2 and day-3 (Epiblastoids-d2, -3), EpiSCs and epiblast cells (E5.5 epiblast, E6.0 epiblast, E6.25 epiblast, E6.5 epiblast). -1#, -2#, and -3# represented different sample repeats. **b** K-Means clustering analysis showed the relationship between Epiblastoids-d2, -3 and EpiLCs.
