# Supplementary material for: Formative pluripotent stem cells show features of epiblast cells poised for gastrulation
Source: Cell Res. 2021 Feb 19;31(5):526–41. doi: 10.1038/s41422-021-00477-x (PMC8089102; doi:10.1038/s41422-021-00477-x)
Supplement: Supplementary file 2 — Supplementary Figure S2 [file 41422_2021_477_MOESM2_ESM.pdf]

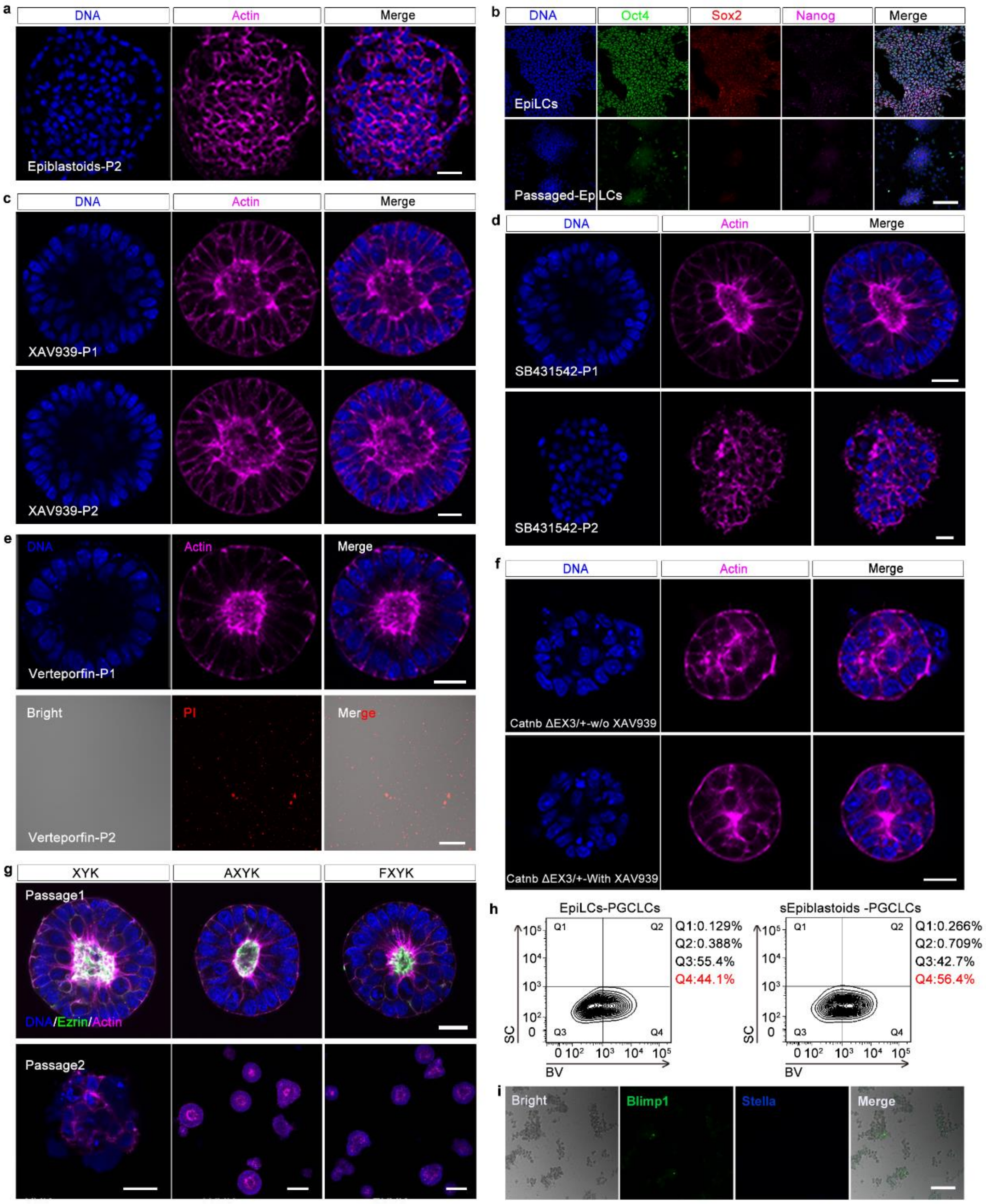

Fig. S2 Screening and capturing the sEpiblastoids.

a Naïve mESCs were cultured in a 3D system with the condition of EpiLC induction and differentiated into Epiblastoids for 3 days. These cells were passaged for propagation (Epiblastoids-P2) under the same condition. The passaged cells were stained with Phalloidin for F-actin (magenta) and Hoechst 33342 for DNA (blue) at day 3. Scale bar, 30 μm. **b** Naïve mESCs were induced into metastable EpiLCs (primary EpiLCs). The induced EpiLCs were propagated in the same medium. The primary EpiLCs (EpiLCs) and propagated EpiLCs (passaged-EpiLCs) were stained with the antibody for Oct4 (green), Sox2 (red) and Nanog (magenta). DNA was stained by Hoechst 33342. Scale bars, 100 μm. **c-e** Naïve mESCs were induced into metastable Epiblastoids and treated with XAV939, SB431542 or Verteporfin (passage 1, P1). These cells were passaged in the same conditions (passage 2, P2) and were

stained with Phalloidin for F-actin (magenta) and Hoechst 33342 for DNA (blue). Scale bars, 20 μm. The PI staining was employed for the dead cells when Epiblastoids were propagated with Verteporfin in (e). Scale bars, 200 μm. **f** *Catnb*$^{(\Delta EX3/+)}$ mESCs were cultured in Epiblastoid medium without/with (upper/ lower panel) XAV939 for 3 days. The cytoskeleton was labelled with Phalloidin (F-actin, magenta) and DNA was stained with Hoechst 33342 (blue). Scale bar, 20 μm. **g** Naïve mESCs were cultured in N2B27 medium supplemented with XAV939, Y27632, and KSR (XYK), with Activin A, XAV939, Y27632, and KSR (AXYK), or with Fgf2, XAV939, Y27632, and KSR (FXYK) for 3 days (Passage1). These cells were passaged in the same conditions (Passage2). The cells were stained for Ezrin with antibody (green), F-actin with Phalloidin (magenta) and DNA with Hoechst 33342 (blue). Scale bars, 20 μm (upper and down-left panel) and 100 μm (down-middle and –right panel). **h** FACS analysis of Blimp1^{+}/Stella^{+} (BV/SC) cells for the aggregates of PGCLCs inducted from BVSC sEpiblastoids and EpiLCs at day 2 after cell differentiation. **i** Blimp1^{+} (green) and Stella^{+} (blue) cells displayed in the PGCLC aggregates of propagated EpiLCs (P2) at day 6 after differentiation. Scale bars, 100 μm.
