# Supplementary material for: Formative pluripotent stem cells show features of epiblast cells poised for gastrulation
Source: Cell Res. 2021 Feb 19;31(5):526–41. doi: 10.1038/s41422-021-00477-x (PMC8089102; doi:10.1038/s41422-021-00477-x)
Supplement: Supplementary file 3 — Supplementary Figure S3 [file 41422_2021_477_MOESM3_ESM.pdf]

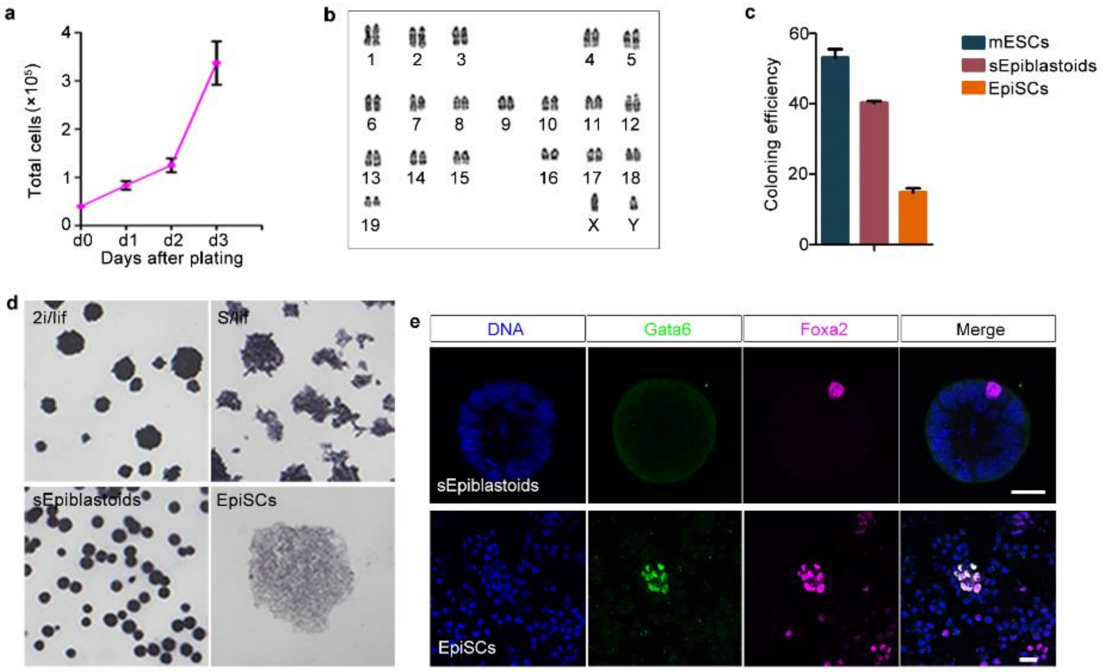

Fig. S3 Further characterization of sEpiblastoids.

a Stabilized Epiblastoids (sEpiblastoids) were passaged and cultured. The cultured cells at different stages were dissociated into single cells and cell number was counted. The cytometry was performed in three independent replicates and cell growth was calculated. Error bars represented SEM. **b** The karyotype analysis was performed for passage 15 sEpiblastoids. **c** Cell colony formation efficiency was calculated for sEpiblastoids, naïve mESCs and EpiSCs. All experiments were repeated three times. Error bars represented SEM. **d** Alkaline phosphatase (AP) staining was performed for naive mESCs (2i/lif), primed mESCs (S/lif), sEpiblastoids and EpiSCs. After staining, the images were captured with microscopy. **e** sEpiblastoids and EpiSCs were fixed and stained with cell lineage marker Gata6 and Foxa2; DNA was stained with Hoechst 33342. Scale bar, 20 μm.
