# Supplementary material for: Formative pluripotent stem cells show features of epiblast cells poised for gastrulation
Source: Cell Res. 2021 Feb 19;31(5):526–41. doi: 10.1038/s41422-021-00477-x (PMC8089102; doi:10.1038/s41422-021-00477-x)
Supplement: Supplementary file 4 — Supplementary Figure S4 [file 41422_2021_477_MOESM4_ESM.pdf]

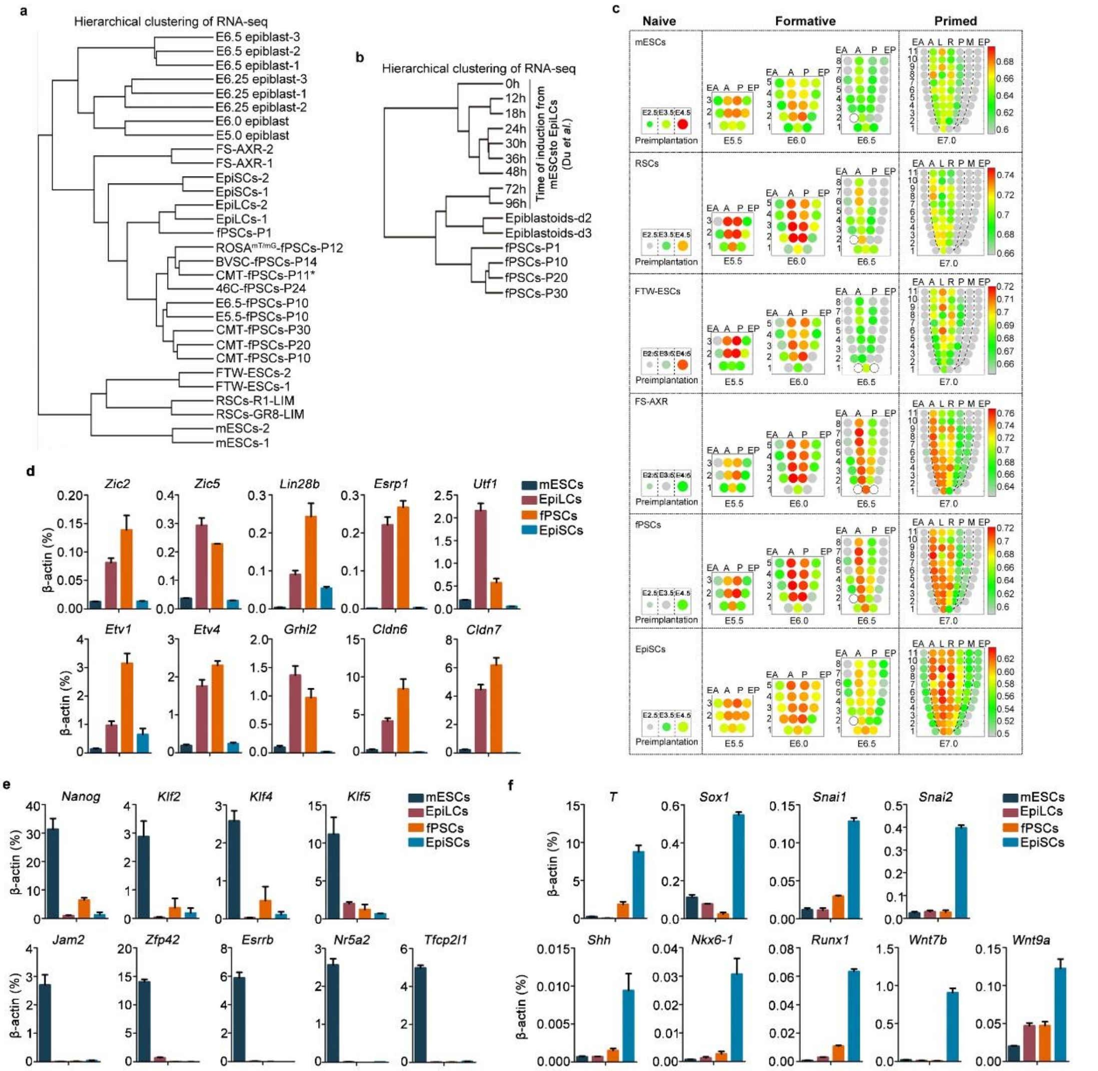

Fig. S4 Further comparison of fPSCs with other pluripotent stem cells.

a, b Hierarchical clustering analysis for RNA-seq data of naïve mESCs, RSCs, FTW-ESCs, EpiLCs, FS-AXR, fPSCs, EpiSCs and pre-/early-gastrula epiblasts[25, 30-32, 42-44]. **c** Deconvolution analysis inferred the developmental stages of mESCs, RSCs, FTW-ESCs, FS-AXR, fPSCs and EpiSCs in the Geo-seq samples as previously reported[45]. Color bar indicated the frequency for each cell type. A, anterior; P, posterior; EA, anterior endoderm; EP, posterior endoderm; L, left lateral; R, right lateral. **d** Quantitative RT-PCR (qRT-PCR) was used to quantify the level of formative genes in mESCs, 48h EpiLCs, fPSCs and EpiSCs. The gene expression level was relative to *β-actin* (100%). Data were mean ± S.D. (n=3). **e** qRT-PCR was used to confirm the expression level of naïve markers (*Nanog*, *Klf2/4/5*, *Jam2*, *Zfp42*, *Esrrb*, *Nr5a2* and *Tfcp2l1*) in mESCs, 48h EpiLCs, fPSCs and EpiSCs. Gene expression level was relative to β-actin (100%). Error bars represented SEM. **f** qRT-PCR was used to confirm the expression level of lineage-biased genes (*T*, *Sox1*, *Snai1/2*, *Shh*, *Nkx6-1*, *Runx1*, *Wnt7b* and *Wnt9a*) in mESCs, 48h EpiLCs, fPSCs and EpiSCs. Gene expression level was relative to β-actin (100%). Error bars represented SEM.
