# Supplementary material for: Formative pluripotent stem cells show features of epiblast cells poised for gastrulation
Source: Cell Res. 2021 Feb 19;31(5):526–41. doi: 10.1038/s41422-021-00477-x (PMC8089102; doi:10.1038/s41422-021-00477-x)
Supplement: Supplementary file 5 — Supplementary Figure S5 [file 41422_2021_477_MOESM5_ESM.pdf]

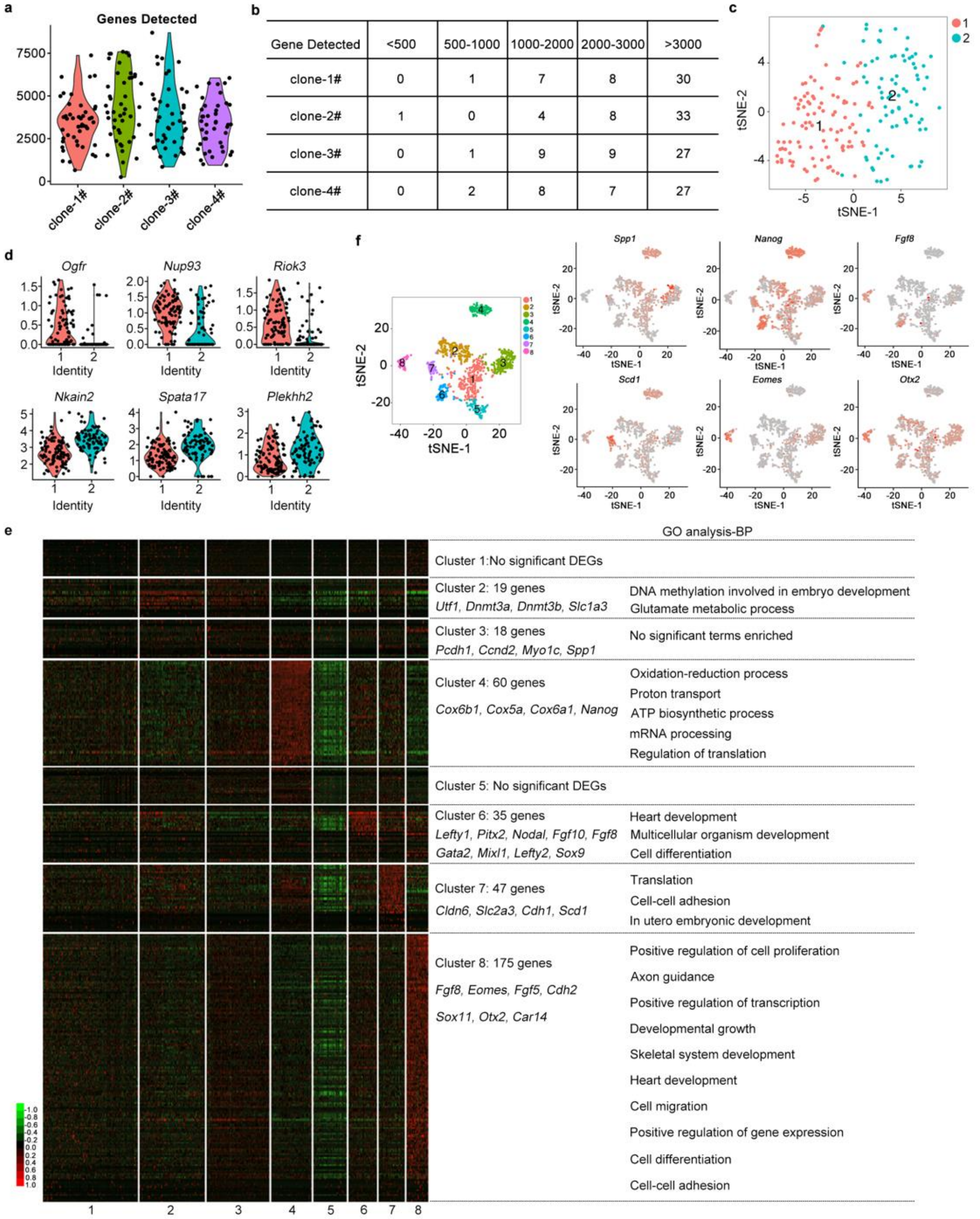

Gene Detected	<500	500-1000	1000-2000	2000-3000	>3000
clone-1#	0	1	7	8	30
clone-2#	1	0	4	8	33
clone-3#	0	1	9	9	27
clone-4#	0	2	8	7	27

Fig. S5 Single-cell transcriptome analysis of fPSCs and data integration.

a-b Data quality control. The number of genes was detected in fPSC single cells of 4 clones. **c** t-SNE plot analysis of single-cell RNA-seq data of fPSCs. Cluster 1 and 2 represented two clusters in fPSCs. **d** Violin plots illustrating the 6 DEGs identified in the cluster 1 or 2 of fPSCs. **e** Data integration and t-SNE analysis of single cells of mESCs (2i/lif, Serum/lif), EpiLCs (48h), RSCs, FS-AXR, fPSCs and EpiSCs[42, 43, 53, 54]. These cells were grouped into 8 clusters and GO analysis was performed for the DEGs in cluster-1 to -8. The gene number and representative genes of DEGs in each cluster were listed in the middle. GO enrichment terms of DEGs were listed in the right. **f** Representative genes highly expressed in cluster-3 (*Spp1*), -4 (*Nanog*), -6 (*Fgf8*), -7 (*Scd1*) and -8 (*Eomes*, *Otx2*).
