# Supplementary material for: Formative pluripotent stem cells show features of epiblast cells poised for gastrulation
Source: Cell Res. 2021 Feb 19;31(5):526–41. doi: 10.1038/s41422-021-00477-x (PMC8089102; doi:10.1038/s41422-021-00477-x)
Supplement: Supplementary file 6 — Supplementary Figure S6 [file 41422_2021_477_MOESM6_ESM.pdf]

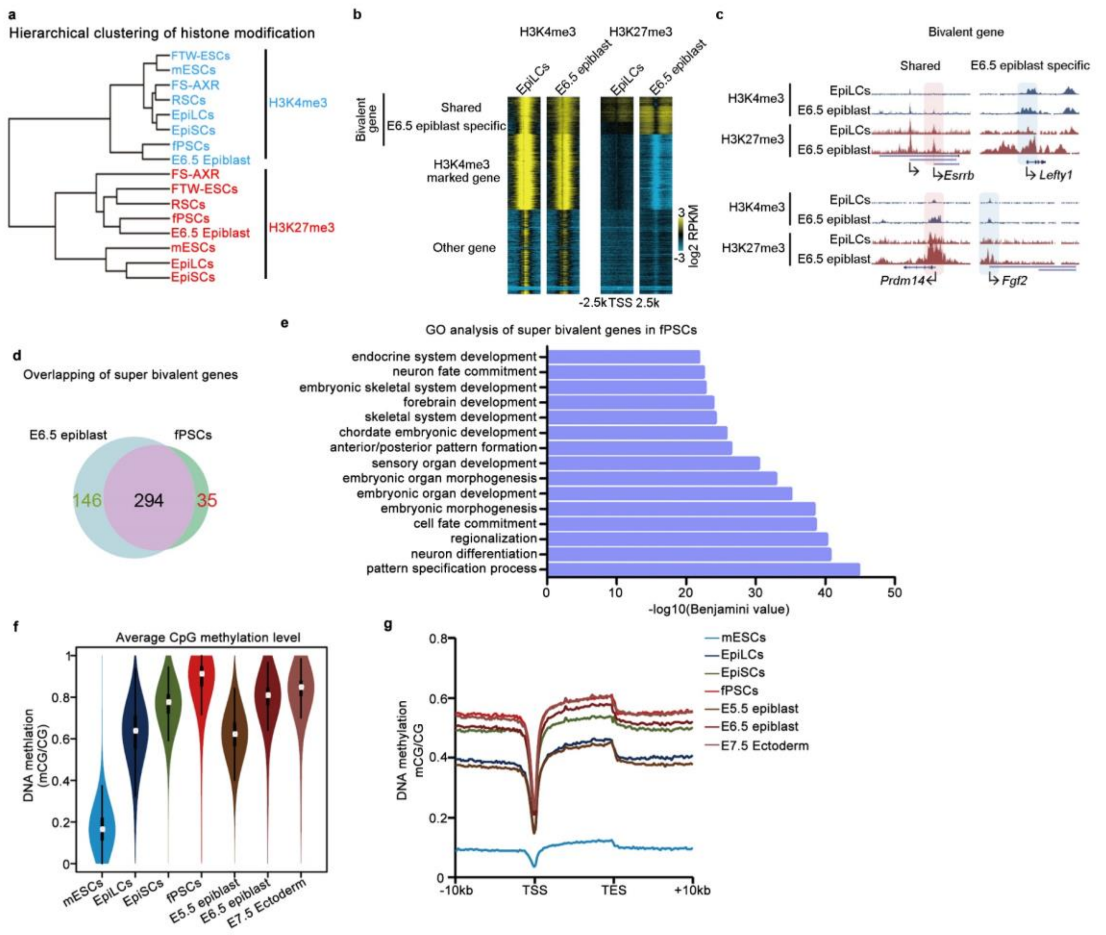

Fig. S6 Histone modification and DNA methylation features of fPSCs.

a Hierarchical clustering of H3K4me3 and H3K27me3 enrichment showing the similarity among mouse ESCs, EpiLCs, RSCs, FTW-ESCs, FS-AXR, EpiSCs, fPSCs and E6.5 epiblasts. The data of ESCs, EpiLCs, RSCs, FTW-ESCs, FS-AXR, EpiSCs and E6.5 epiblasts were obtained from previous reports[42-44, 56, 57]. **b** Heatmaps showing H3K4me3 and H3K27me3 enrichment at bivalent genes, H3K4me3 marked gene and other gene in EpiLCs and E6.5 epiblast. **c** Snapshots showing the distribution of H3K4me3 and H3K27me3 at selected promoters of specific genes in EpiLCs and E6.5 epiblast. Promoters with H3K27me3 in both EpiLCs and E6.5 epiblast, and E6.5 epiblast specific promoters enriched for H3K27me3 are shaded by different colors. **d** The Venn diagram showing the number of overlapping super bivalent genes (pink) in fPSCs (pink and yellow green) and E6.5 epiblast (pink and light green). **e** GO analysis was performed for the super bivalent genes in fPSCs to identify the enriched signaling pathways in these cells. **f** The average level of CpG methylation of mESCs, EpiLCs, EpiSCs, fPSCs and E5.5/6.5/7.5 epiblasts. The WGBS profiles of mouse ESCs, EpiLCs, EpiSCs and E5.5/6.5/7.5 epiblasts was obtained from previous studies[58-61]. **g** The average level of DNA methylation at TSS adjacent area (± 10kb) among mESCs, EpiLCs, fPSCs, EpiSCs and E5.5/6.5/7.5 epiblasts. TSS, transcription start site; TES, transcription end site.
