# Supplementary material for: Formative pluripotent stem cells show features of epiblast cells poised for gastrulation
Source: Cell Res. 2021 Feb 19;31(5):526–41. doi: 10.1038/s41422-021-00477-x (PMC8089102; doi:10.1038/s41422-021-00477-x)
Supplement: Supplementary file 7 — Supplementary Figure S7 [file 41422_2021_477_MOESM7_ESM.pdf]

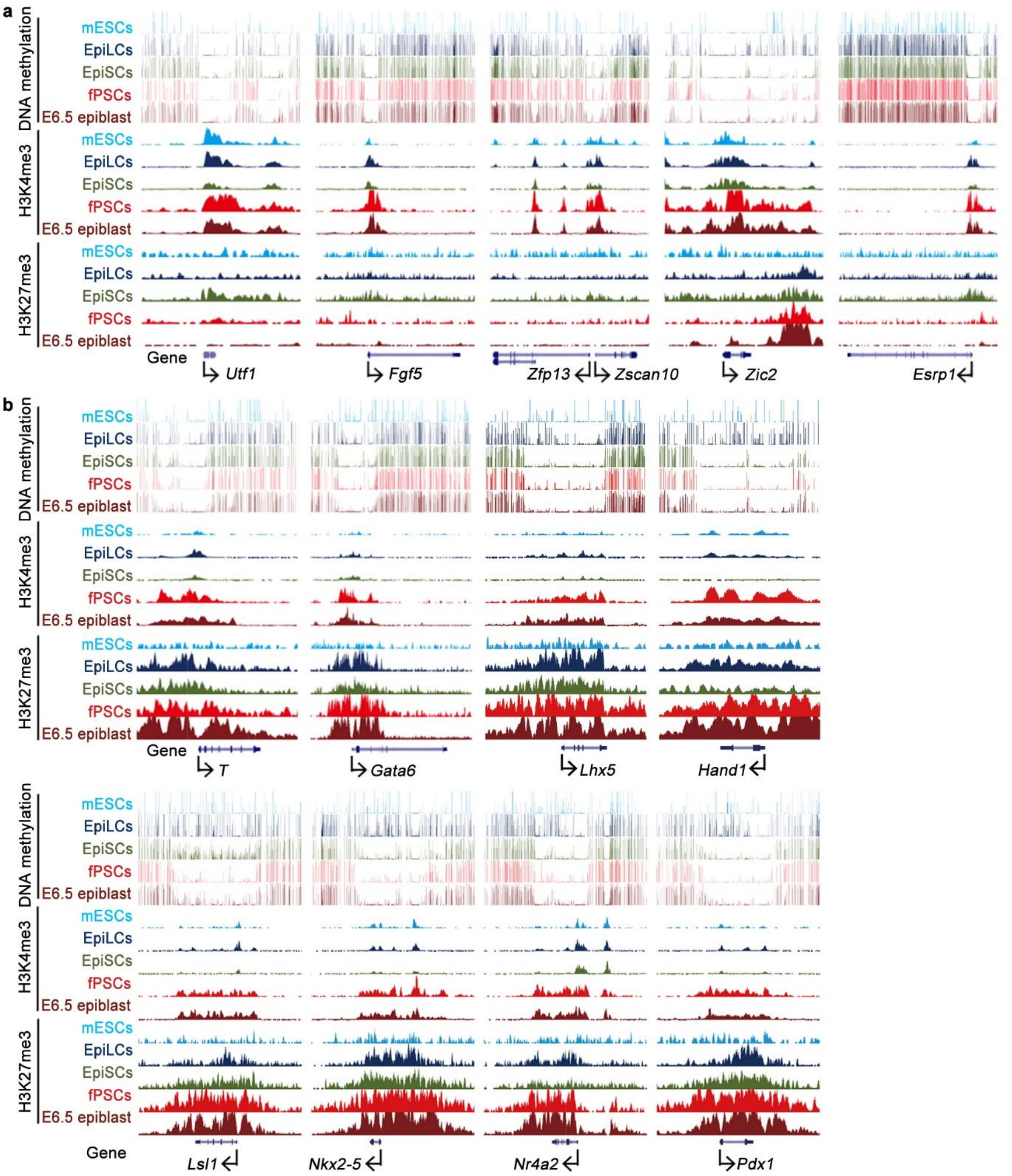

Fig. S7 Epigenetic features of specific formative and lineage genes in fPSCs.

a UCSC Genome Browser views showing DNA methylation and H3K4me3 /H3K27me3 enrichment at the promoters of formative genes (*Utf1*, *Fgf5*, *Zfp13*, *Zscan10*, *Zic2* and *Esrp1*) among mESCs, EpiLCs, fPSCs, EpiSCs and E6.5 epiblast. The data of mESCs, EpiLCs, EpiSCs and E6.5 epiblast was previously reported[56-61]. **b** Snapshots of DNA methylation and H3K4me3/H3K27me3 enrichment at the promoters of lineage specific genes (*T*, *Gata6*, *Lhx5*, *Hand1*, *Isl1*, *Nkx2-5*, *Nr4a2* and *Pdx1*) in mESCs, EpiLCs, fPSCs, EpiSCs and E6.5 epiblast.
