# Supplementary material for: Formative pluripotent stem cells show features of epiblast cells poised for gastrulation
Source: Cell Res. 2021 Feb 19;31(5):526–41. doi: 10.1038/s41422-021-00477-x (PMC8089102; doi:10.1038/s41422-021-00477-x)
Supplement: Supplementary file 8 — Supplementary Figure S8 [file 41422_2021_477_MOESM8_ESM.pdf]

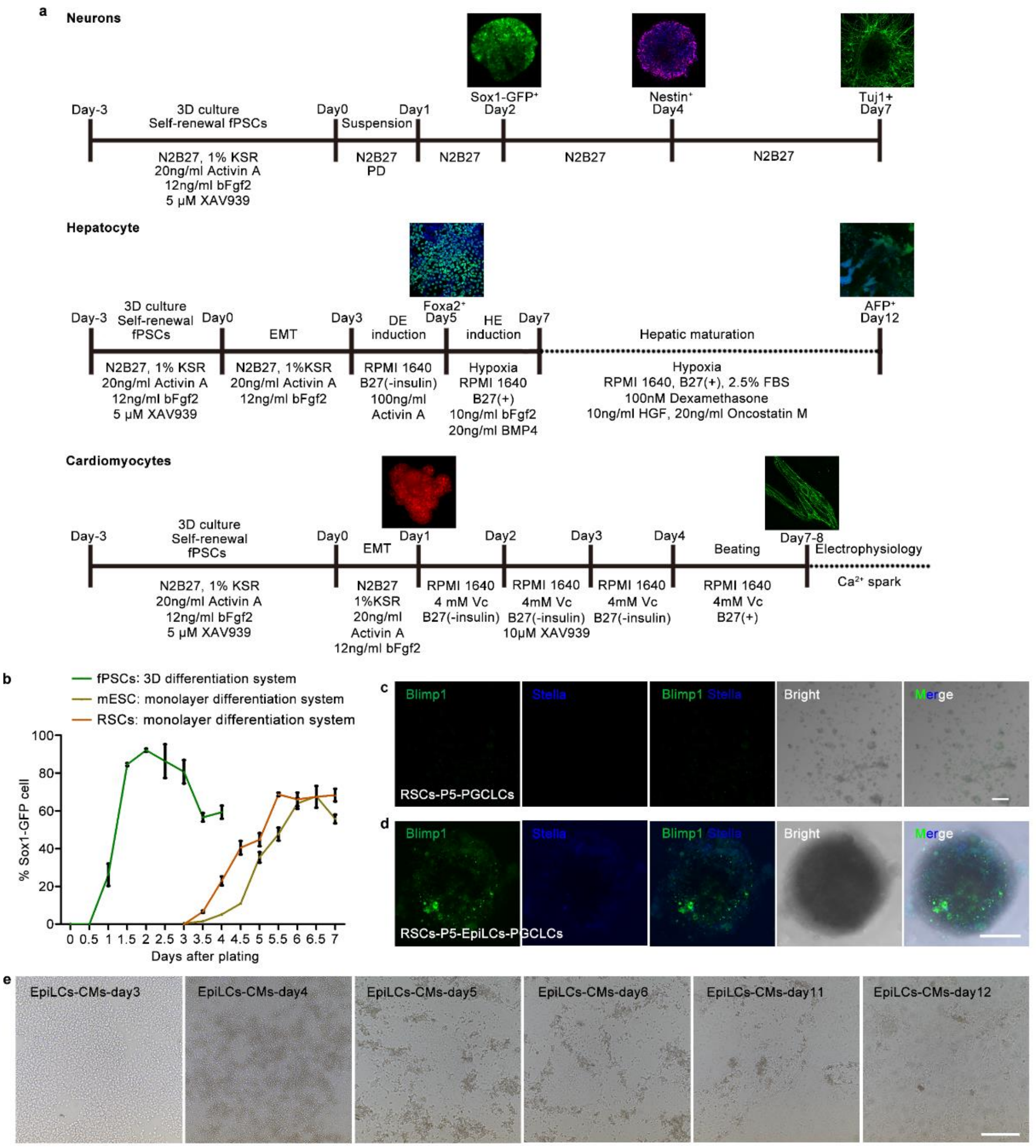

Fig. S8 Differentiation of fPSCs, mESCs, EpiLCs and RSCs in vitro.

a The protocols used for the induction of fPSCs into three germ layers, including: ectoderm (neuronal-like cells), endoderm (hepatic-like cells) and mesoderm (cardiomyocyte-like cells). **b** The fPSCs derived from 46C ESCs differentiated as in (A). Sox1-GFP positive cells were determined at the interval of 12 hrs by flow cytometry. The percentage of Sox1-GFP^{+} inducted from the fPSCs, RSCs and mESCs was calculated. Each time point was performed in 3 independent experiments. Error bars was represented SEM. **c** Blimp1^{+} (green) and Stella^{+} (blue) cells displayed in the PGCLC aggregates of RSCs-P5 at day 6 after cell digestion. Scale bars, 100 μm. **d** Blimp1^{+} (green) and Stella^{+} (blue) cells displayed in the PGCLC aggregates of EpiLCs derived from RSCs-P5 at day 6 after cell digestion. Scale bars, 100 μm. **e** The differentiation of EpiLCs to cardiomyocytes from day 3 to day 6 and day 11 to day 12. Scale bar, 50 μm.
