# Supplementary material for: Formative pluripotent stem cells show features of epiblast cells poised for gastrulation
Source: Cell Res. 2021 Feb 19;31(5):526–41. doi: 10.1038/s41422-021-00477-x (PMC8089102; doi:10.1038/s41422-021-00477-x)
Supplement: Supplementary file 9 — Supplementary Figure S9 [file 41422_2021_477_MOESM9_ESM.pdf]

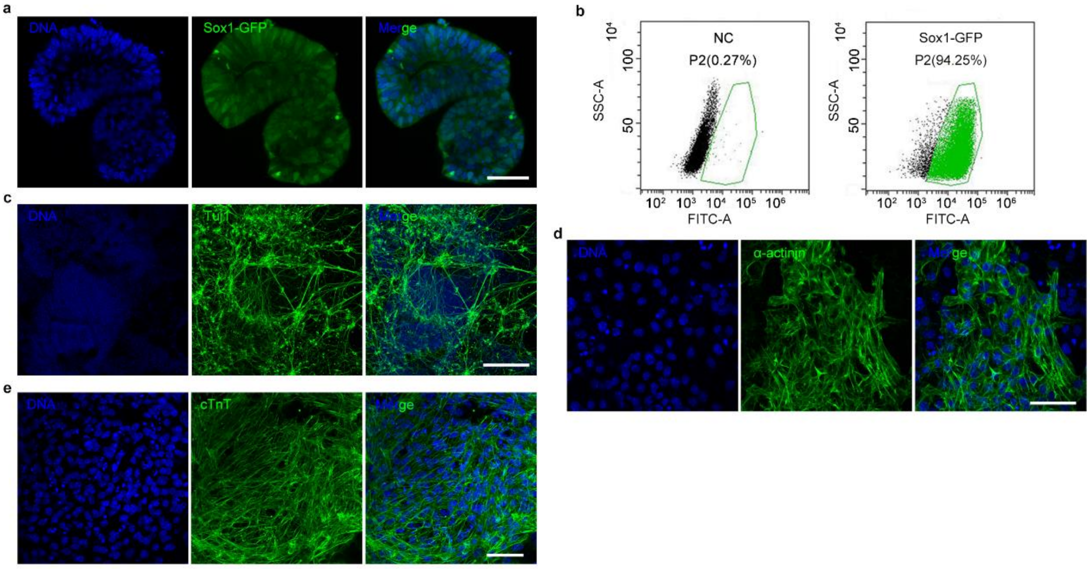

Fig. S9 Characterization of the differentiation capacity of cryopreserved fPSCs.

a Sox1-GFP$^+$ cells obtained from the cryopreserved 46C-fPSCs and DNA stained with Hoechst 33342 (blue). Scale bar, 50 μm. **b** The proportion of Sox1-GFP$^+$ cells differentiated from the cryopreserved 46C-fPSCs. The cryopreserved 46C-fPSCs was induced into Sox1-GFP$^+$ cells and analyzed by FACS. The cryopreserved CMT-fPSCs was induced in the same conditions and was served as negative control (NC). **c** Tuj1$^+$ neurons obtained from the cryopreserved 46C-fPSCs and DNA stained with Hoechst 33342 (blue). Scale bar, 200 μm. **d, e** The cardiomyocytes derived from the cryopreserved 46C-fPSCs were stained with the antibody for α-actinin or cTnT (green). DNA was stained with Hoechst 33342 (blue). Scale bar, 50 μm.
