# Supplementary material for: Formative pluripotent stem cells show features of epiblast cells poised for gastrulation
Source: Cell Res. 2021 Feb 19;31(5):526–41. doi: 10.1038/s41422-021-00477-x (PMC8089102; doi:10.1038/s41422-021-00477-x)
Supplement: Supplementary file 10 — Supplementary Figure S10 [file 41422_2021_477_MOESM10_ESM.pdf]

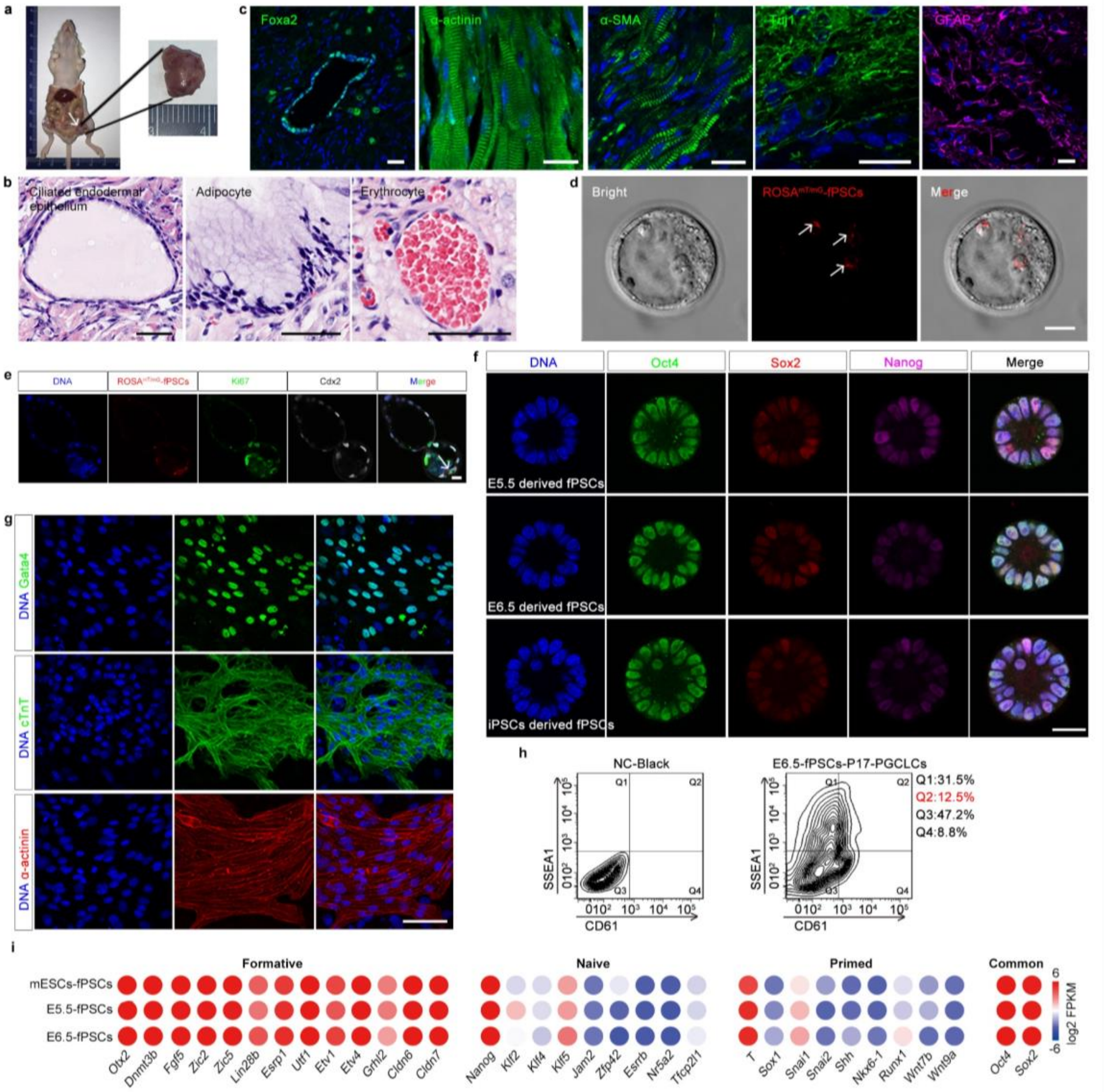

**Fig. S10 Differentiation of fPSCs in vivo and in vitro.**

**a** The morphology of teratoma generated from CMT-fPSCs. **b** The paraffin sections of teratoma generated from CMT-fPSCs were subjected to Haematoxylin and eosin (HE) staining. Left panel: Ciliated endodermal epithelium; middle panel: Adipocyte; right panel: Erythrocyte. Scale bars, 50 μm. **c** The paraffin sections of teratoma generated from CMT-fPSCs were stained with the specific antibodies of three germ layer markers (left panel, endoderm marker Foxa2; middle panel, mesoderm muscle marker α-actinin and α-SMA; right panel, ectoderm neural marker Tuj1 and GFAP). DNA was stained with Hoechst 33342. Scale bar, 20 μm. **d** *ROSA*$^{mT/mG}$-fPSCs were injected into mouse blastocysts and cultured for 6 hours. The images were obtained by fluorescent confocal microscope. Scale bar, 20 μm. **e** *ROSA*$^{mT/mG}$-fPSCs were injected into mouse blastocysts and cultured for 24 hours. The embryos were fixed and stained with the antibody for Ki67 (green), and Cdx2 (white). DNA was labeled with Hoechst 33342. Scale bar, 20 μm. **f** The expression pattern of Oct4 (green), Sox2 (red) and Nanog (magenta) in the fPSCs obtained from E5.5, 6.5 epiblasts and iPSCs. Nuclear DNA was stained with Hoechst 33342 (blue). Scale bars, 50 μm. **g** Cardiomyocyte-like cells were induced from the fPSCs derived mouse epiblasts. The differentiated fPSCs were stained with the specific antibodies for

cardiomyocyte markers (Gata4, cTnT and α-actinin). DNA was stained with Hoechst 33342. Scale bar, 50 μm. **h** FACS analysis of SSEA1 and CD61 (integrin-β3) double-positive cells for the aggregates of PGCLCs generated from the E6.5 epiblast derived fPSCs (E6.5-fPSCs-P17). NC (negative control), the experiment without the antibodies. **i** The expression of selected formative, primed, naïve and common pluripotent markers in the fPSCs derived from mESCs (mESCs-fPSCs), in vivo E5.5 (E5.5-fPSCs) and E6.5 epiblast (E6.5-fPSCs).
