# Supplementary material for: Formative pluripotent stem cells show features of epiblast cells poised for gastrulation
Source: Cell Res. 2021 Feb 19;31(5):526–41. doi: 10.1038/s41422-021-00477-x (PMC8089102; doi:10.1038/s41422-021-00477-x)
Supplement: Supplementary file 16 — Supplementary Table S6 [file 41422_2021_477_MOESM16_ESM.pdf]

Tables S6. Primer information used for qRT-PCR

Genes	Forward Primer（5'-3'）	Reverse Primer（5'-3'）
Otx2	GGAAGAGGTGGCACTGAAAAT	CTGTTGGCGGCACTTAGCT
Dnmt3b	CCTGCCCGCAAAGGTTTA	CCAGAAGAATGGACGGTTGTC
Sox4	CTCCTCGTCCTCTTCCTCCTC	TGCCCAGGGACATGCTCT
Fgf5	GAAATATTTGCTGTGTCTCAGGG	TAAATTTGGCACTTGCATGG
Zic2	CAAGTCCTACACGCATCCCA	GTGGACGACTCGTAGCCAGA
Zic5	CGAGGCTGTGATAAATCGTACA	CACCGACGAGTAGCCAAGAG
Lin28b	CAAAGGGAGATAGGTGGAGACG	CGAGACCACCACAGTTGTAGCA
Esrp1	CAAGGAGTTGCTGGGTAAAAGA	GTTGGAAGTGGAATCAGAGGG
Utf1	GCTGGCTGCGGAAAATGA	GCAGAGTGTCGGTGCTCGTAA
Etv1	CTGAAGACGGACATGGAACG	GTTGTAGGGGTGAGGGTTGC
Etv4	GCTCTGAAGGCTGAGTTTGACC	GCCAGTGAGTTCTGGGAGGTAG
Grhl2	CCAGAACAAGGGATAGGAAGCC	GGAAACGAGGCACAGCAGAG
Cldn6	CTGGGTGGAGGGCTACTATGC	GAGGGTCCCCGAGAATGTG
Cldn7	TAATGGTGGTGTCCCTGGTG	TTCTTCGCTTTGTCATCTCCC
Fgf8	GTGGAGACCGATACTTTTGGAA	GTGGAGACCGATACTTTTGGAA
Nanog	CCTCAGCCTCCAGCAGATGC	CCGCTTGCACTTCACCCTTTG
Klf2	CTATCTTGCCGTCCTTTGCC	TTTAGGTCCTCATCCGTGCC
Klf4	TGCTCCCGTCCTTCTCCAC	CCTCACGCCAACGGTTAGTC
Klf5	TCCCGATAGACAAGCTGAGATG	AACTGGCAGAGTGGCAGGTAA
Jam2	GGACTATCATAAGGCAAATGGG	GGAGGTAGTCTTCTTTGGGGTT
Rex1	CCGTGTAACATACACCATCCG	TGCACTCTAGGTATCCGTCAGG
Esrrb	GGGATGCTGAAGGAAGGTGT	TTAGCAGGTGGGGAAATCG
Nr5a2	TGCTGGACTACACGGTTTGC	TGCCTGCTTGCTGATTGC
Tfcp2l1	GGCTGACGGAACTACGGGA	GAGATAGGTCAGTGCTTGAAATGG
T	CCAGAATGAGGAGATTACAGCC	TGGTCGTTTCTTTCTTTGGC
Sox1	AGACTTCGAGCCGACAAGAG	AACTGTGCAAACAGGTGCAG
Snai1	CGATGAGGACAGTGGCAAAA	CAGGGAAGGCGATGAAGG
Snai2	TCCAAGGATCACAGTGGTTCA	AAACTTCTCAGCTTCGATGGC
Shh	CAATCTGCAACGGAAGCG	CAGGTGCCAATGTGGTAGAGC
Nkx6-1	GGGACTTCGGAGAATGAGGA	ACCGCTCGATTTGTGCTTT
Rnux1	GCGGCTCAGTGAATTGGAG	GGTTAAAGGCAGTGGAGTGGT
Wnt7b	TGCCTTCACCTATGCCATCA	TTGTAGTAGCCTTGCTTCTCCC
Wnt9a	GATTTGCGAGCCCGAGTG	ACGGTGCAGGAGCCAGAC
β-actin	ATGCTCCCCGGGCTGTAT	CATAGGAGTCCTTCTGACCCATTC

Supplementary Movies

Movie S1. The occurrence of EMT in Epiblastoids beyond 3 Days

Movie S2. Beating colonies differentiated from fPSCs

Movie S3. Ca2+ release and spark in the cardiomyocyte-like cells
